# Dissecting cell-type-specific roles of androgen receptor in prostate homeostasis and regeneration through lineage tracing

**DOI:** 10.1038/ncomms14284

**Published:** 2017-01-23

**Authors:** Qing Xie, Yueli Liu, Tao Cai, Corrigan Horton, Joshua Stefanson, Zhu A. Wang

**Affiliations:** 1 Department of Molecular, Cell and Developmental Biology, University of California, Santa Cruz, California 95064, USA; 2 Sequencing Center, National Institute of Biological Sciences, Beijing 102206, China

## Abstract

Androgen signals through androgen receptor (AR) to influence prostate development and cancer. How stromal and epithelial AR regulate prostate homeostasis remains unclear. Using genetic lineage tracing, we systematically investigated the role of cell-autonomous AR in different prostate epithelial cell types. Here we show that AR is dispensable for basal cell maintenance, but is cell-autonomously required for the luminal differentiation of rare basal stem cells. In contrast, AR deletion in luminal cells alters cell morphology and induces transient over-proliferation, without affecting androgen-mediated luminal cell survival or regeneration. However, AR is selectively required for the maintenance of daughter cells produced by castration-resistant *Nkx3.1*-expressing luminal stem cells (CARNs). Notably, Pten loss can override AR-loss effects in both basal and luminal compartments to initiate tumours. Our data reveal distinct cell-type-specific roles of epithelial AR in orchestrating prostate homeostasis, and question the notion that epithelial AR serves as a tumour suppressor in early cancer initiation.

## Abstract (web summary)

Androgen receptor is an important regulator of prostate development and cancer. In this study, the authors use genetic lineage tracing in mice to clarify the role of AR in different prostate epithelial cells.

The steroid hormone, androgen, plays critical roles in prostate development and cancer progression through its nuclear receptor, androgen receptor (AR)^1^,^2^. However, the specific functions of AR in those processes remain elusive, and investigations into this question are complicated by the dynamic and heterogeneous expression pattern of AR in different prostate cell types through time. The prostate gland consists of stromal tissues that mainly include interstitial fibroblasts and smooth muscle cells, as well as an epithelium that includes rare neuroendocrine cells and two major cell types, namely, basal cells that express cytokeratin (CK) 5, p63 and low levels of AR, and luminal cells that express CK18, Nkx3.1 and high levels of AR^3^. Classic tissue recombination experiments showed that stromal AR, but not epithelial AR, is essential for prostate developmental growth and morphogenesis through paracrine signals^4^,^5^,^6^. Later mouse genetic studies using AR conditional knockout in stromal fibroblasts and/or smooth muscle cells also reported reduced prostate size, decreased epithelial proliferation and impaired histology^7^,^8^,^9^,^10^; although the tumour-suppressing/promoting role for stromal AR during prostate cancer progression is still debated^11^,^12^. In contrast, in the prostate epithelium, AR function has traditionally been thought to primarily regulate the expression of androgen-dependent secretory proteins^1^,^13^. Recently, several Cre lines were used to ablate AR in the mouse prostate epithelium during postnatal development. While some studies reported a tumour-suppressing role for epithelial AR, others reported varied and sometimes contradictory phenotypes concerning the behaviours of basal and luminal cells^14^,^15^,^16^,^17^. To date, the function of AR in the adult prostate epithelium, particularly at the resolution of specific adult epithelial cell types, remains unclear. Acquiring such knowledge will be crucial for our understanding of prostate homeostasis and cancer initiation.

Recent mouse lineage-tracing studies from multiple groups have provided a clearer picture of the cell lineage relationship in the normal prostate epithelium *in vivo*. Whereas basal cells serve as multipotent stem cells to generate luminal and neuroendocrine cells during prostate development^18^, their stem cell activities become gradually restricted as the prostate organ matures^19^. In adult prostate homeostasis, basal and luminal cells are largely two self-sustained lineages, with very low proliferation in both compartments and basal-to-luminal cell differentiation occurring only occasionally^19^,^20^. When androgen is deprived through castration, the prostate will regress as the majority of luminal cells undergo cell apoptosis while basal cells are largely unaffected. Re-administration of androgen will lead to luminal cell regeneration and prostate growing back to normal size. Such androgen-mediated prostate regression–regeneration can be repeated for multiple cycles in rodents^21^. Lineage-tracing analyses showed that luminal cell regeneration is primarily due to the proliferation of the remaining luminal cells that survive androgen deprivation^20^,^22^, although rare basal stem cell (BSC) activities also minimally contribute^19^. Whether cell-autonomous AR in those cell types drives regeneration in response to androgen is unknown. In addition, while all luminal cells in the hormonal-intact prostate express the transcription factor Nkx3.1, in the regressed prostate, Nkx3.1-expressing luminal cells (named CARNs) are rare, and they were shown to behave as a type of luminal stem cell that can produce both luminal and basal cells during regeneration^23^. Since *Nkx3.1* is a downstream target gene of AR^24^,^25^, the role of AR in CARNs awaits to be investigated.

Deletion of the tumour suppressor gene *Pten* in the mouse prostate epithelium has served as a highly relevant model for studying human prostate cancer^26^. Under this oncogenic condition, basal, luminal and CARN cells all can serve as the cell of origin for prostate cancer^19^,^20^,^23^,^27^. Recently, it was shown that epithelial AR in general is not required for the initiation and progression of *Pten*-null prostate cancer^28^. However, whether basal- and luminal-specific ARs play different roles in tumour formation is unknown. Here, we set out to investigate the role of AR in different prostate epithelial cell lineages in the context of adult prostate homeostasis, androgen-mediated prostate regression–regeneration and tumour initiation. Through cell-type-specific ablation of AR coupled with lineage-tracing analyses, our data demonstrate distinct AR functions in adult basal and luminal cells, and uncover its essential roles in the multipotent capability of rare stem cells in both compartments.

## Results

### Heterogeneous expression and dispensability of basal cell AR

**Figure 1:**
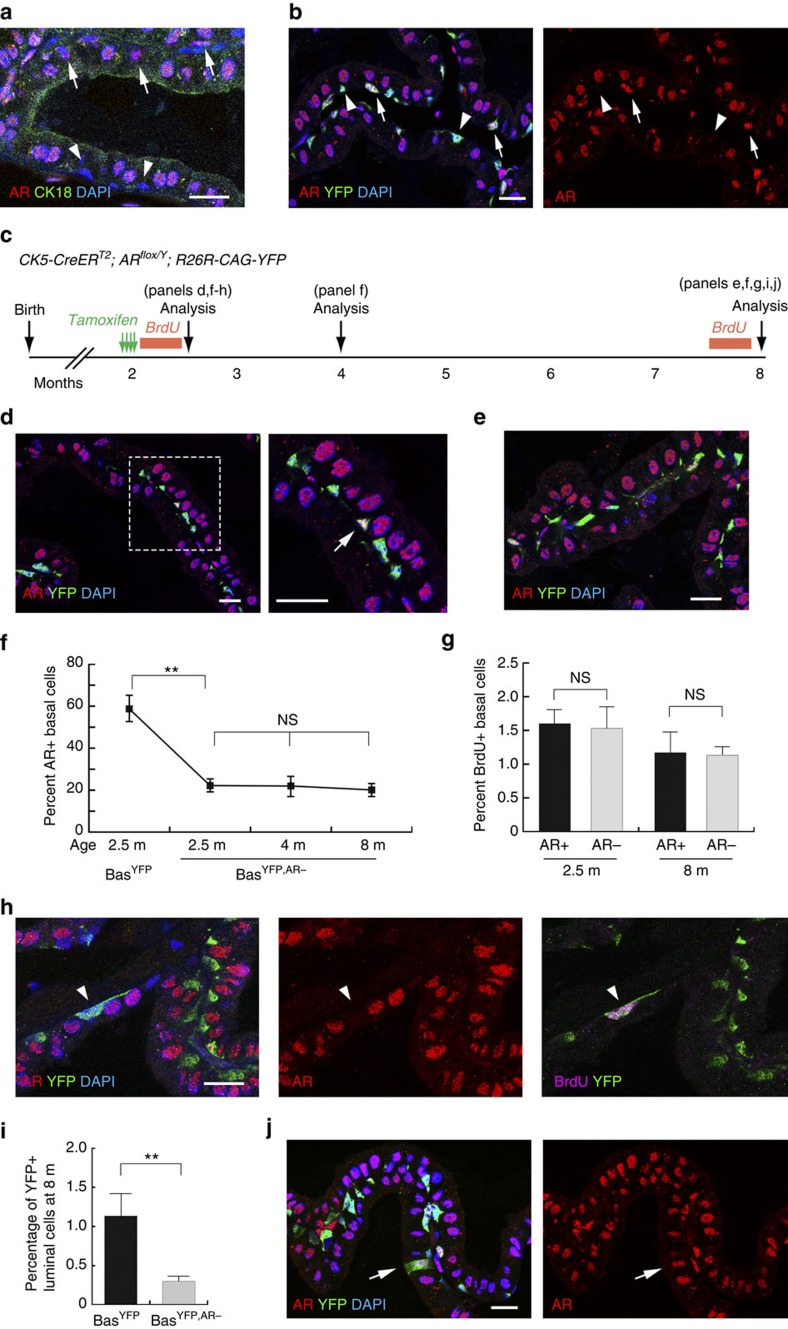
**Lineage analysis of AR**^−^ **and AR**^**+**^ **basal cells in prostate homeostasis.** (**a**) AR expression in all the luminal cells and the presence of AR^+^ (arrows) and AR^−^ (arrowheads) basal cells. (**b**) Lineage-marking of basal cells by CAG-YFP is highly penetrant and facilitates quantitation of AR^+^ (arrows) and AR^−^ basal cells (arrowheads). (**c**) Lineage-tracing strategy in prostate homeostasis of Bas^YFP,AR−^ mice. (**d**) Efficient deletion of AR in basal cells at 2 weeks post induction. (**e**) Normal tissue morphology and absence of AR in the majority of basal cells at 6 months after induction. (**f**) Percentage of AR^+^ basal cells decreased significantly after AR deletion and remained stable thereafter in homeostasis. **P<0.001 by *t*-test. (**g**) AR^+^ and AR^−^ basal cells have the same proliferation rates in BrdU incorporation assays of Bas^YFP,AR−^ mice during homeostasis. NS by *t*-test. (**h**) Representative image of BrdU staining analysed at 2.5 m. Arrowhead points to an AR^−^ basal cell that is BrdU^+^. (**i**) Percentage of YFP^+^ luminal cells decreased significantly in Bas^YFP,AR−^ mice at 6 months post induction compared to Bas^YFP^ mice. **P<0.0001 by *χ*^2^ test. (**j**) The rare YFP^+^ luminal cells were always AR^+^ (arrow). Scale bars correspond to 20 μm. Error bars correspond to one s.d.

AR has been considered absent or expressed at very low levels in adult prostate basal cells, but highly expressed in luminal cells. Our immunofluorescence (IF) staining of adult mouse prostate confirmed strong nuclear AR expression in all luminal cells (Fig. 1a), and interestingly, revealed its expression to be heterogeneous in the basal layer, as strong AR nuclear staining was randomly present in a subset of basal cells (Fig. 1a). For lineage analysis of basal cells, we used the previously characterized inducible basal-specific driver *CK5-CreER*^*T2*^(refs 19, 29). For better visualization and quantitation of basal AR, we tamoxifen-induced *CK5-CreER*^*T2*^*; R26R-CAG-YFP/+* (denoted Bas^YFP^) mice, in which almost all of the basal cells (98.7%, *n*=8,807/8,921, three animals analysed) can be marked by a CAG promoter-driven enhanced YFP^30^, with 58.7% of the marked cells positive for AR staining (Fig. 1b,f; Supplementary Table 1).

To test whether AR is functionally significant in adult basal cells, we conditionally deleted the AR gene in them by tamoxifen induction of 8-week old *CK5-CreER*^*T2*^*; AR*^*flox/Y*^*; R26R-CAG-YFP/+* (denoted Bas^YFP,AR−^) male mice and performed lineage tracing (Fig. 1c). The *AR*^*flox*^ allele deletes exon 2 upon induction, leading to disruption of the sequence encoding the DNA binding domain and yielding a non-functional transcript harbouring a frame shift and premature stop codon^31^,^32^. We found basal AR deletion to be efficient but not fully penetrant, as the percentage of YFP^+^ basal cells that were AR^+^ significantly decreased to 22.2% in the anterior prostate (AP) lobes 2 weeks after induction (three animals analysed, *P*<0.001 by *t*-test, Fig. 1d,f; Supplementary Table 1). In the subsequent tracing period of up to 8 months of age, no abnormality of the prostatic tubule structure or morphological changes of either basal or luminal cells were observed (Fig. 1e), and the percentage of AR^+^ basal cells remained stable at the reduced ratio (Fig. 1f; Supplementary Table 1). Furthermore, cleaved Caspase 3 staining revealed no elevation of apoptosis in the epithelium, and both BrdU incorporation assays (Fig. 1c) and Ki67 staining showed that the proliferation rates in AR^+^ and AR^−^ basal cells were similar (Fig. 1g,h; Supplementary Fig. 1; Supplementary Table 1). AR deletion was less efficient in the ventral prostate and dorsal–lateral prostate lobes than in the AP, but the proportions of AR^+^ and AR^−^ basal cells also remained stable throughout the tracing period (Supplementary Fig. 2). Taken together, we conclude that AR expression in adult prostate basal cells is dispensable for their normal homeostasis.

### AR is cell-autonomously required for BSC differentiation

Prostate epithelial cells are normally lineage-restricted in the adult organ, with rare basal cells occasionally undergoing luminal differentiation *in vivo*^19^,^20^. Consistent with previous reports, we found that, in the AP lobes of 8-month-old Bas^YFP^ mice that had undergone lineage tracing (Fig. 1c), 1.1% of YFP^+^ cells were luminal (*n*=59/5,177, three animals analysed; Fig. 1i; Supplementary Table 1). In contrast, this basal-to-luminal differentiation ratio significantly decreased to 0.31% (*n*=17/5,545, three animals analysed, *P*<0.0001 by *χ*^2^ test) in lineage-traced 8-month-old Bas^YFP,AR−^ mice (Fig. 1i; Supplementary Table 1). Importantly, all of the rare YFP^+^ luminal cells were AR^+^ (Fig. 1j; Supplementary Fig. 2b, right panel), suggesting they were derived from basal cells that escaped AR deletion. In fact, we never observed even a single AR-negative YFP^+^ luminal cell from Bas^YFP^ or Bas^YFP,AR−^ mice (*n*=0/76, six animals analysed, Supplementary Table 1). These data demonstrate that AR is cell-autonomously required for the bipotentiality of rare adult BSCs in organ homeostasis.

**Figure 2:**
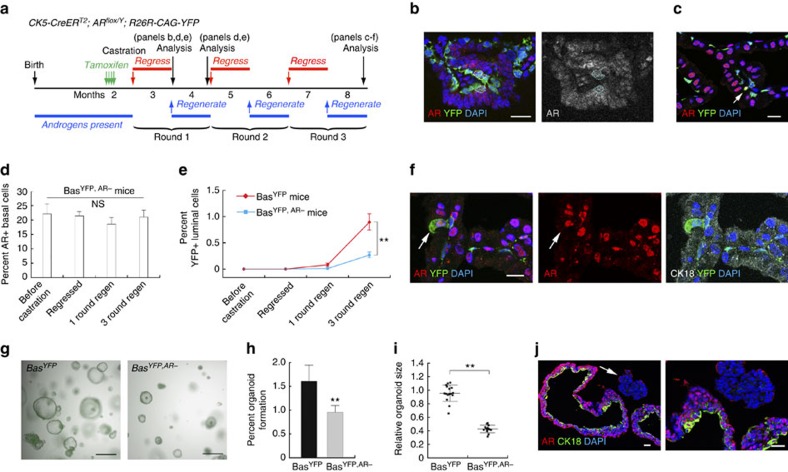
**Cell-autonomous requirement of AR in BSCs** ***in vivo*** **and in organoid culture.** (**a**) Lineage-tracing strategy in serial regression/regeneration of Bas^YFP^ and Bas^YFP,AR−^ mice. (**b**) Most basal cells (marked by YFP) were AR^−^ after induction and castration in the regressed prostate. Circles show AR^+^ basal cells that escaped AR deletion. (**c**) Normal cell morphology and few YFP^+^ luminal cells after three rounds of regression–regeneration. Most basal cells remained AR^−^. Arrow points to an AR^+^ basal cells. (**d**) Percentage of AR^+^ basal cells stayed constant in Bas^YFP,AR−^ mice during serial regression–regeneration. NS by *t*-test. (**e**) Percentage of luminal cells among total YFP^+^ cells gradually increased in Bas^YFP^ mice during serial regeneration, but was significantly reduced in Bas^YFP,AR−^ mice. **P<0.0001 by *χ*^2^ test. (**f**) Rare YFP^+^ luminal cells after 3 rounds of regression–regeneration in Bas^YFP,AR−^ mice were always AR^+^ (arrow). (**g**) White field and YFP overlay images showing morphology and abundance of organoids derived from basal cells of Bas^YFP^ and Bas^YFP,AR−^ mice. (**h**) Bar graph comparing organoid formation efficiencies from 16 wells of seeded Bas^YFP^ cells and 14 wells of seeded Bas^YFP,AR−^ cells. **P<0.001 by *t*-test. (**i**) Comparison of average organoid size (normalized) per well for 16 wells of seeded Bas^YFP^ cells and 14 wells of seeded Bas^YFP,AR−^ cells. **P<0.001 by *t*-test. (**j**) IF section staining showing lack of hollow lumen and little basal-to-luminal differentiation in an AR^−^ basal organoid (arrow and zoom-in on the right) compared to adjacent wild-type basal-derived organoids. Scale bars in **b**,**c**,**f**,**j** correspond to 20 μm, and in **g** to 0.5 mm. Error bars correspond to one s.d.

We next studied the behaviours of AR^−^ basal cells in the context of androgen-mediated prostate regression–regeneration. Bas^YFP,AR−^ mice were induced at 8-weeks of age and then castrated 2 weeks later (Fig. 2a). We found that 21.5% of the basal cells in the regressed prostate remained AR^+^ (Fig. 2b,d; Supplementary Table 2), a ratio similar to that found before castration. During subsequent serial regression–regeneration of up to three rounds, the percentage of AR^+^ basal cells remained constantly around 20% (Fig. 2c,d; Supplementary Table 2). Similar results were obtained in the ventral prostate and dorsal–lateral prostate lobes (Supplementary Fig. 3). Therefore, androgen levels do not appear to affect the relative balance between AR^+^ and AR^−^ basal cells. In lineage-traced Bas^YFP^ mice that had undergone three rounds of prostate regression–regeneration, we found that 0.89% of the YFP^+^ cells were luminal (*n*=71/7,990, three animals analysed, Fig. 2e; Supplementary Table 2), consistent with the previous finding that rare BSCs can give rise to a small proportion of luminal cells during serial regeneration^19^. In contrast, the luminal YFP^+^ ratio significantly decreased to 0.27% in Bas^YFP,AR−^ mice (*n*=23/8,585, three animals analysed, *P*<0.0001 by *χ*^2^ test, Fig. 2e; Supplementary Table 2). Again, all of the rare YFP^+^ luminal cells were AR^+^ (Fig. 2f; Supplementary Fig. 3b, right panel), and AR-negative YFP^+^ luminal cells were never found in Bas^YFP^ or Bas^YFP,AR−^ mice (*n*=0/102, 12 animals analysed, Supplementary Table 2). Therefore, cell-autonomous AR is also required for BSCs to differentiate into luminal cells in prostate regeneration.

To corroborate these lineage-tracing findings, we investigated the role of AR in BSCs using the recently developed organoid technique^33^,^34^,^35^. YFP^+^ basal cells were flow-sorted from tamoxifen-induced Bas^YFP^ and Bas^YFP,AR−^ mice, respectively, and 10,000 cells were seeded per well and cultured using a previous protocol^36^. Basal cells isolated from Bas^YFP^ mice yielded a significantly greater number of organoids than those from Bas^YFP,AR−^ mice (Fig. 2g,h, *P*<0.001 by *t*-test), and they also had significantly larger average sizes and more branching (Fig. 2g,i, *P*<0.001 by *t*-test). Since the seeded Bas^YFP,AR−^ cells were a mixed population of wild type and AR-deleted basal cells, IF staining of individual organoids showed that, compared with wild-type controls, organoids grown from AR^−^ basal cells lacked the hollow lumen and had little basal-to-luminal cell differentiation as revealed by CK18 staining (Fig. 2j). Therefore, these *in vitro* data also support our conclusions drawn from *in vivo* lineage tracing experiments.

### AR^−^ luminal cells expand transiently with altered morphology

**Figure 3:**
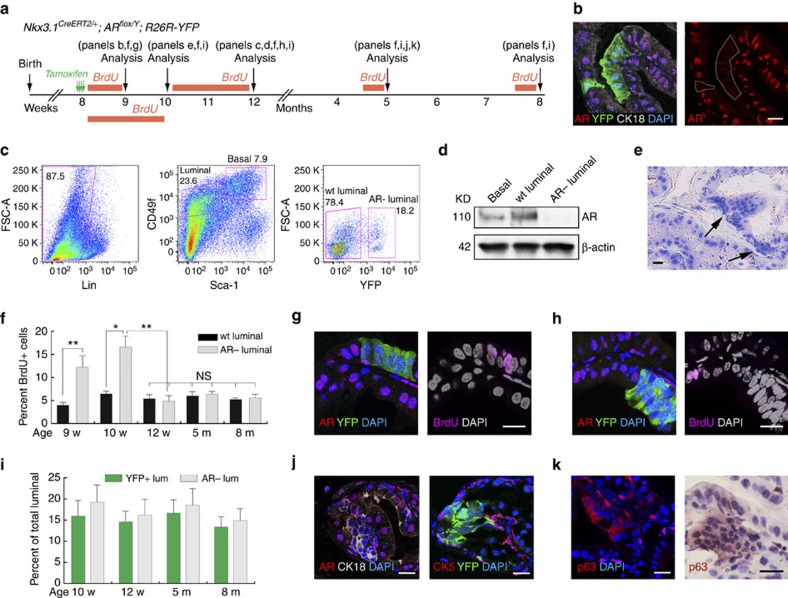
AR loss in luminal cells induces a transient over-proliferation and alters cell morphology. (**a**) Lineage-tracing strategy in prostate homeostasis of Lum^YFP,AR−^ mice. (**b**) Representative IF staining image showing simultaneous deletion of AR and marking by YFP in luminal cells at 1 week post induction. (**c**) FACS plot showing the gate drawn for obtaining basal, wild-type luminal and AR^−^ luminal cells from induced Lum^YFP,AR−^ mice. (**d**) Western blot of AR protein from flow-sorted cell populations. (**e**) H&E staining showing clusters of compact cells (arrows) at 2 weeks post induction. (**f**) Quantitation of wild-type and AR^−^ luminal cell proliferation rates using BrdU incorporation assays shown in **a**, showing a transient (1–2 weeks) over-proliferation in AR^−^ luminal cells. *P<0.01, **P<0.001 by *t*-test. (**g**,**h**) Representative IF staining images showing many AR^−^ luminal cells were BrdU^+^ at 1 week post induction (**g**) and relatively few were BrdU^+^ at 4 weeks post induction (**h**). (**i**) Quantitation of the percentage of YFP^+^ or AR^−^ luminal cells during homeostasis showing no significant difference by *t*-test for different time points since 2 weeks post induction. (**j**) IF staining of tissues at 3 months post induction showing enhanced CK18 expression (left) and no detectable CK5 expression (right) in AR^−^/YFP^+^ luminal cell clusters. (**k**) Cytoplasmic p63 expression was enhanced in AR^−^ luminal cell clusters by both IF (left) and IHC (right) staining using two antibodies. Scale bars correspond to 20 μm. Error bars correspond to one s.d.

Since AR is strongly expressed in the nuclei of all adult luminal cells, we next investigated the effects of luminal AR loss-of-function using the luminal-specific driver *Nkx3.1*^*CreERT2/+*^(ref. 23). *Nkx3.1*^*CreERT2/+*^*; AR*^*flox/Y*^*; R26R-YFP/+* (denoted Lum^YFP,AR−^) mice were tamoxifen-induced at 8 weeks of age and analysed through adult homeostasis (Fig. 3a). IF staining revealed that YFP fluorescence can reliably indicate AR deletion, since almost all YFP^+^ cells (98.7%, *n*=1,698/1,720, four animals analysed) were also AR^−^, while 84.8% of AR^−^ cells (*n*=1,698/2,002, four animals analysed) were also YFP^+^ (Fig. 3b, Supplementary Fig. 4a). To further validate the cell type specificity of AR expression, we utilized an established flow-sorting protocol^37^,^38^ to isolate basal cells (Lin^−^CD49f^hi^Sca-1^+^), wild-type luminal cells (Lin^−^CD49f^low^Sca-1^−^YFP^−^) and AR-deleted luminal cells (Lin^−^CD49f^low^Sca-1^−^YFP^+^) from induced Lum^YFP,AR−^ mice (Fig. 3c). Western blot analysis of sorted cells confirmed that AR expression is higher in wild-type luminal cells than basal cells, but is absent in AR-deleted luminal cells (Fig. 3d).

During the tracing period, we did not observe any luminal cell sloughing into the lumen or elevation of luminal cell apoptosis by cleaved Caspase 3 staining, suggesting AR is not cell-autonomously required for luminal cell survival. Strikingly, within 2 weeks after induction the AR^−^ luminal cells appeared as condensed cell clusters that were morphologically distinguished from wild-type luminal cells, as these cells were more compact and usually in stacked layers (Fig. 3e). BrdU incorporation assays (Fig. 3a) and Ki67 staining both showed that AR^−^ luminal cells experienced a burst of fast proliferation during the first 1–2 weeks post induction (Fig. 3f,g; Supplementary Fig. 4b,f; Supplementary Table 3), but after this brief period, their proliferation rates decreased back to normal levels that were similar to wild-type luminal cells (Fig. 3f,h; Supplementary Fig. 4c,f; Supplementary Table 3). Such initial over-proliferation explains the origin of the observed condensed AR^−^ luminal cell clusters, and its transient nature agrees with our lineage-tracing data, which showed that from 2 weeks post induction onwards, the ratio of AR^−^ or YFP^+^ luminal cells did not increase through time (Fig. 3i; Supplementary Table 3). Notably, no difference in overall basal cell proliferation was observed during the tracing period (Supplementary Fig. 5).

To further characterize the AR^−^ luminal cells morphologically, we performed staining with different cell markers. AR^−^ luminal cells retained some luminal features, as they showed enhanced CK18 expression compared with wild-type ones, and no detectable CK5 expression (Fig. 3j). Interestingly, both IF and IHC staining using two different antibodies showed that expression of another basal marker p63 was enhanced in the cytoplasm of AR^−^ luminal cells (Fig. 3k), suggesting they may resemble intermediate cells. Furthermore, Nkx3.1 expression in these cells was abolished (Supplementary Fig. 4d), suggesting it is a downstream target gene of cell-autonomous AR^24^,^25^. αPKC, a polarity marker that is normally expressed on the apical side of luminal cells, and E-Cadherin, which is normally expressed on the lateral sides^39^, were both highly expressed all around the surface of AR^−^ luminal cells compared with adjacent wild-type counterparts (Supplementary Fig. 4e), indicating a disruption of normal cell polarity.

### Gene expression profiling analyses of AR^−^ luminal cells

**Figure 4:**
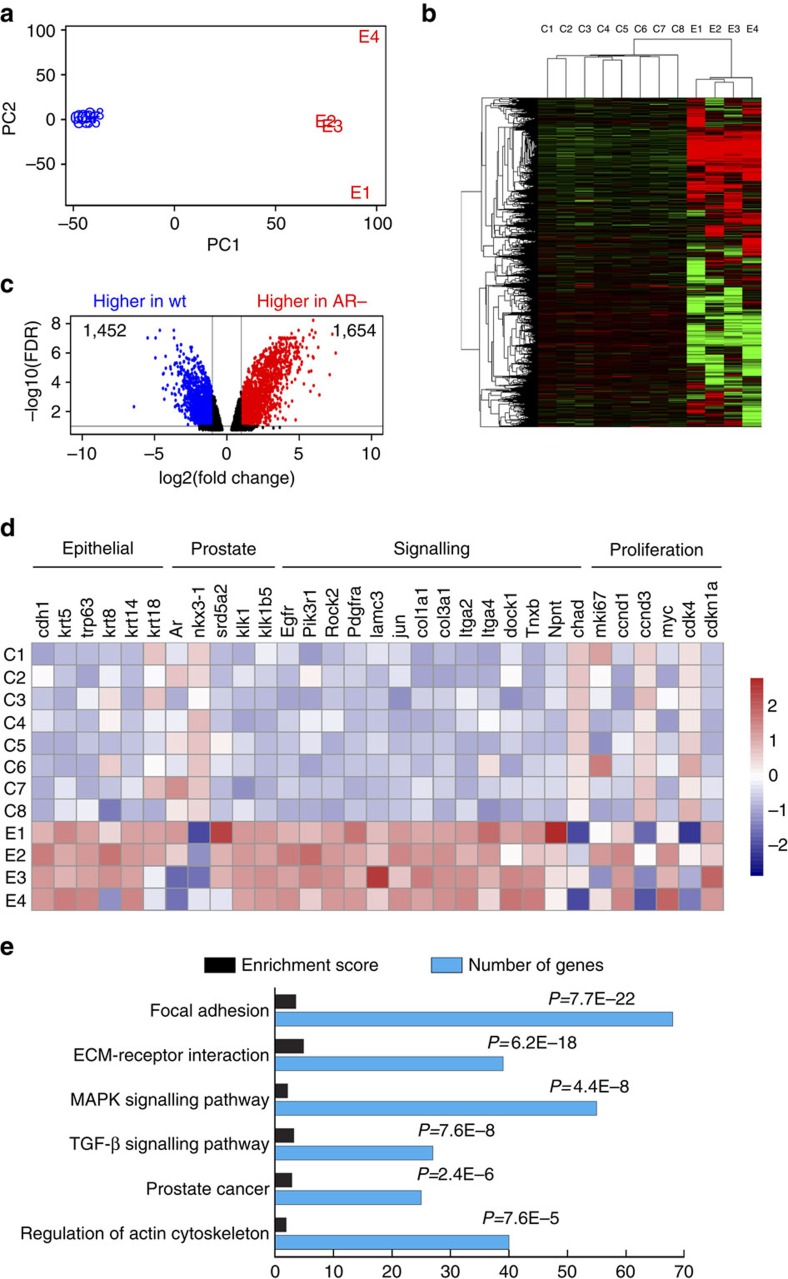
**Gene expression profiling analyses of AR**^−^ **luminal cells.** (**a**) Scatter-plot of the two main components from a Principal Component Analysis of control (wild-type, blue points) and experimental (AR^−^, red points) luminal samples based on 9,238 genes after filtering out too lowly or too highly expressed genes, capturing 44.5% (dimension 1) and 16.6% (dimension 2) of the data variability. (**b**) Unsupervised hierarchical clustering analysis showing good separation of control samples (C1–C8) and AR^−^ luminal samples (E1–E4). (**c**) Volcano plot showing 1,654 genes are upregulated and 1,452 genes are downregulated in AR^−^ luminal cells (FDR<0.1 and fold change >2). (**d**) Expression levels of selected genes in different samples showing a general upregulation of epithelial markers and various cell signalling genes in AR^−^ luminal cells, and mixed expression pattern changes for proliferation markers. (**e**) DAVID GO analysis showing the most enriched pathways in AR^−^ luminal cells (FDR<0.1) and the number of genes in each pathway.

Next, we performed RNA-seq analysis of wild type and AR^−^ luminal cells to compare their gene expression profiles. We isolated wild-type and AR^−^ luminal cells by flow-sorting of YFP^+^ cells from *Nkx3.1*^*CreERT2/+*^*; R26R-YFP/+* (denoted Lum^YFP^, control) and Lum^YFP,AR−^ (experimental) mice 1 month after induction, respectively (Supplementary Fig. 6a). Cytospin analysis of flow-sorted cells showed that 97.6% of YFP^+^ cells from the experimental mice were AR^−^, while 99.1% of YFP^+^ cells from the control mice were AR^+^ (Supplementary Fig. 6b). RNA-seq was performed for eight control and four experimental samples (all were biological replicates). Principal components analysis (PCA) and unsupervised hierarchical clustering analysis demonstrated that the independent samples within each group were consistent and that the control and experimental groups were well separated (Fig. 4a,b). A total of 1,654 genes were upregulated and 1,452 genes were downregulated in AR^−^ luminal cells compared with the wild-type control (Fig. 4c; Supplementary Data 1,2; false discovery rate (FDR) <0.1, and fold change >2). As expected, both RNA-seq data and our quantitative real-time PCR results showed that the AR target gene *Nkx3.1* was downregulated in AR^−^ luminal cells (Fig. 4d; Supplementary Fig. 6c). Notably, both basal and luminal epithelial cell marker genes (*cdh1*, *trp63*, *krt8*, *krt14*, *krt18*) were upregulated (Fig. 4d; Supplementary Fig. 6c), indicating AR^−^ luminal cells may have molecular features of intermediate cells. Furthermore, genes involved in cell proliferation (*mki67*, *ccnd1*, *ccnd3*, *myc*, *cdk4*, *cdkn1a*) showed mixed or insignificant expression level changes (Fig. 4d; Supplementary Fig. 6c), consistent with our finding that AR^−^ luminal cells at this stage are transitioning away from a hyper-proliferative state. DAVID GO analysis^40^ identified 12 enriched molecular pathways in AR^−^ luminal cells (FDR <0.1; Supplementary Data 3), with the most notable ones implicated in cell-matrix adhesions, MAPK and TGF-β signalling pathways, prostate cancer and cytoskeleton regulation (Fig. 4e). Collectively, these data suggest that AR^−^ luminal cells are primarily altered in cell morphology, and share some molecular signatures with prostate cancer cells.

### Luminal cell-autonomous AR is dispensable for regeneration

**Figure 5:**
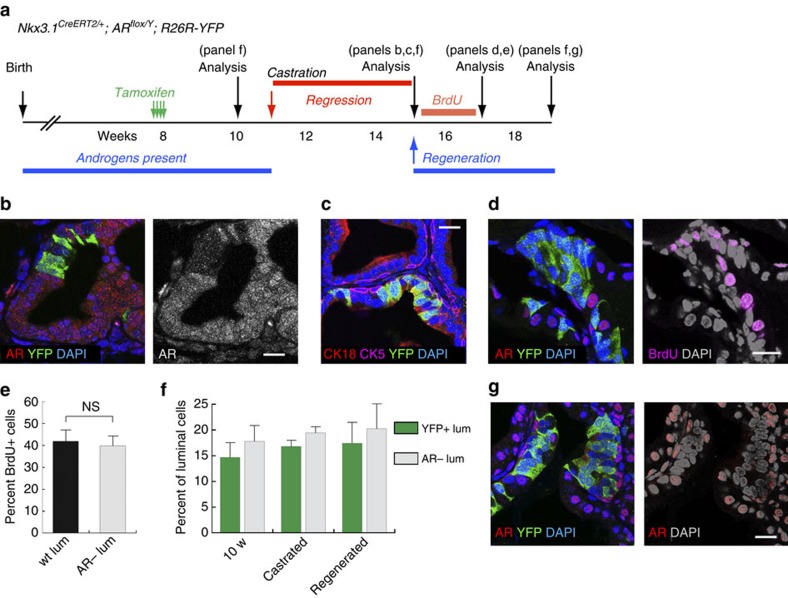
Cell-autonomous AR is dispensable for luminal cell regeneration. (**a**) Lineage-tracing strategy for average luminal cells during prostate regeneration in Lum^YFP,AR−^ mice. (**b**) IF staining showing AR^−^ luminal cells were present in regressed prostate and marked by YFP. (**c**) CK5, CK18 and YFP triple staining showing marked cells in the regressed prostate remained strictly luminal. (**d**) Representative image showing high proliferation in both AR^+^ and AR^−^ luminal cells in a BrdU incorporation assay during prostate regeneration. (**e**) Quantitation of cell proliferation in the BrdU incorporation assay showing no difference by *t*-test between AR^+^ and AR^−^ luminal cells. (**f**) The percentages of YFP^+^ or AR^−^ luminal cells among total luminal cells during the course of prostate regression–regeneration remained constant by *t*-test. (**g**) YFP^+^AR^−^ luminal cell clusters were present and had compact cell phenotypes after prostate regeneration. Scale bars correspond to 20 μm. Error bars correspond to one s.d.

Classic tissue recombination experiments showed that during organogenesis AR^+^ mesenchyme cells could promote AR^−^ epithelial cells to grow and generate prostate tissues through paracrine signals^4^,^5^,^6^. However, whether luminal cell-autonomous AR is required for adult prostate regeneration remains unknown. To test this, we induced AR^−^ luminal cells in 8-week old hormonal-intact Lum^YFP,AR−^ mice, and then lineage-traced them in the processes of castration and androgen re-administration (Fig. 5a). We found that, after castration, almost all of the YFP^+^ cells (97.8%, *n*=529/541, three animals analysed) in the regressed prostate were AR^−^, whereas the unmarked luminal cells showed diffusive AR staining, which would be expected in the absence of androgen (Fig. 5b; Supplementary Fig. 7a). These YFP^+^ cells remained strictly luminal as revealed by CK5 and CK18 staining (Fig. 5c; Supplementary Fig. 7a, right panel). Importantly, the percentage of luminal cells that were YFP^+^ or AR^−^ did not change before and after castration (Fig. 5f; Supplementary Table 4), indicating that AR-expression levels in luminal cells do not affect the susceptibility/resistance of these cells to androgen deprivation. BrdU incorporation assay was performed for 12 days following testosterone pump implantation (Fig. 5a), and we detected robust proliferation in both YFP^+^AR^−^ and YFP^−^AR^+^ luminal cells (Fig. 5d; Supplementary Fig. 7b), with rates being similar between the two populations (Fig. 5e; Supplementary Table 4). Ki67 staining performed at 4 days after pump implantation also confirmed this result (Supplementary Fig. 8). Notably, in the fully regenerated prostate, the percentage of YFP^+^ or AR^−^ luminal cells remained unchanged (Fig. 5f; Supplementary Fig. 7d; Supplementary Table 4), and they showed the ‘compaction' phenotype (Fig. 5g; Supplementary Fig. 7c). These data demonstrate that cell-autonomous AR is dispensable for average regressed luminal cells to regenerate. Although the data do not directly prove the case, they strongly support the hypothesis that androgen acts on stromal AR to mediate adult luminal cell regeneration through paracrine signals.

### AR is selectively required for CARN stem cell activities

**Figure 6:**
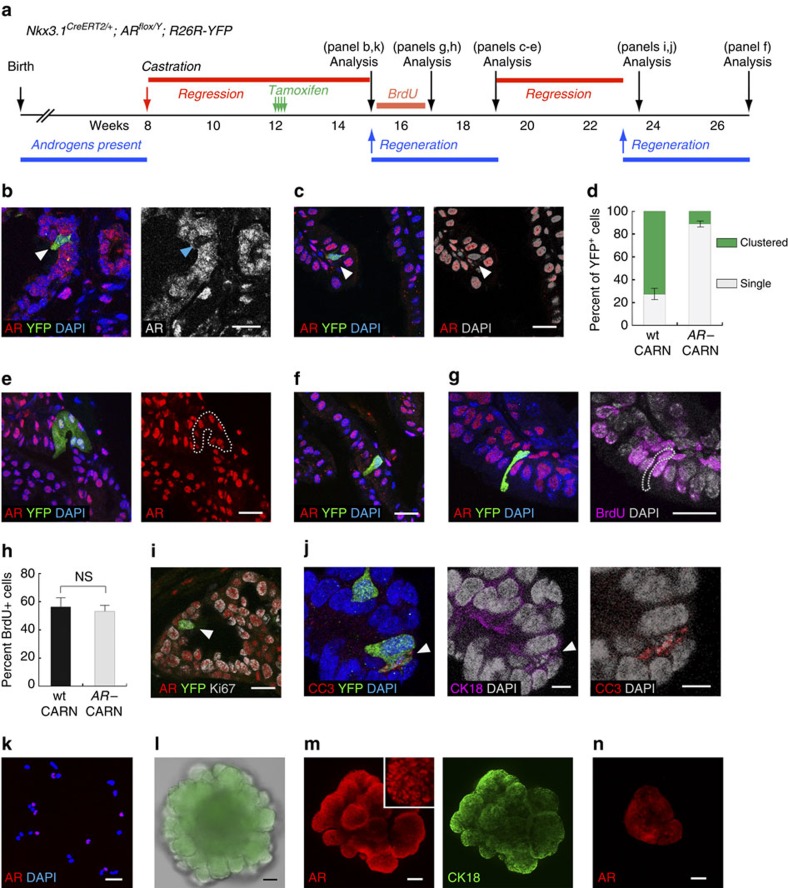
**AR is selectively required for CARN stem cell differentiation**. (**a**) Lineage-tracing strategy for CARNs during serial prostate regression–regeneration in Lum^YFP,AR−^ mice. (**b**) Lineage-marked AR^−^ CARNs (arrowhead) survived in the regressed prostate after AR deletion. (**c**) Isolated single YFP^+^AR^−^ cells (arrowhead) were present after one round of regeneration. (**d**) Quantitation of the proportions of clustered and single YFP^+^ cells derived from wild-type CARNs (Lum^YFP^) and AR^−^ CARNs (Lum^YFP,AR−^) after one round of regeneration showing the deficiency of AR^−^ CARNs to generate cell clusters by fisher's exact test. (**e**) Regenerated cells in rare YFP^+^ cell clusters in Lum^YFP,AR−^ mice were AR^+^. (**f**) Regenerated YFP^+^AR^−^ cells remained as single isolated cells after two rounds of regression–regeneration. (**g**) Representative image showing AR^−^ CARNs were proliferating in a BrdU incorporation assay during prostate regeneration. (**h**) Quantitation of cell proliferation in the BrdU incorporation assay during prostate regeneration showing no difference between wild-type and AR^−^ CARNs by *t*-test. (**i**) Representative image showing an AR^−^ CARN cell (arrowhead) stained positive for Ki67 4 days after androgen re-administration. (**j**) Cleaved caspase 3 (CC3), YFP and CK18 triple staining showing that the daughter cell (arrowhead) of an AR^−^ CARN is apoptotic. (**k**) IF staining of cytospin preparation of sorted CARN cells from Lum^YFP,AR−^ mice showing a mixture of AR^+^ and AR^−^ CARNs. (**l**) Representative white field and YFP overlay image showing morphology of CARN organoid. (**m**) Representative *in situ* IF image of an organoid derived from AR^+^ CARN. A zoom-in portion shown in inset. (**n**) IF staining of the only organoid that contained a mixture of AR^+^ and AR^−^ cells. Scale bars in **b**,**c**, **e**–**g**,**i**–**k** correspond to 20 μm, and in **l**–**n** to 100 μm. Error bars correspond to one s.d.

In the regressed prostate, rare castration-resistant *Nkx3.1*-expressing cells (CARNs) were shown to be a type of stem cell that can produce luminal and, to a lesser extent, basal cells during prostate regeneration^23^. To determine the uniqueness of CARNs in prostate regeneration compared with average regressed luminal cells, we next investigated whether their stem cell activities are dependent on AR. Lum^YFP,AR−^ mice were first castrated at 8 weeks of age and then induced 4 weeks later to mark CARNs and simultaneously delete AR in them (Fig. 6a). Consistent with the study of wild-type CARNs^23^, we found that, in the regressed prostate, 1.0% of the luminal cells (*n*=74/7,786, three animals analysed) were marked by YFP (Fig. 6b). Most of these YFP^+^ cells (82.4%, *n*=61/74, three animals analysed) were AR^−^ (Fig. 6b), suggesting deletion of AR in CARNs was efficient and did not affect their survival. AR^−^ CARNs did not express *Nkx3.1* (Supplementary Fig. 9a), indicating cell-autonomous AR directly activates *Nkx3.1* expression in normal CARNs. Upon completion of prostate regeneration, we detected isolated single YFP^+^AR^−^ cells (Fig. 6c). YFP^+^ cell clusters (defined as >3 adjacent cells) in the regenerated prostate were rare, in contrast to results obtained from wild-type CARNs in Lum^YFP^ mice (Fig. 6d; Supplementary Table 5). Notably, the cells in those rare clusters were AR^+^ (Fig. 6e), suggesting that they were derived from wild-type CARNs that escaped AR deletion. The same phenotypes were also observed after two rounds of regression–regeneration (Fig. 6f). Surprisingly, the failure of AR^−^ CARNs to produce cell clusters was not due to a defect in CARN cell proliferation, because we found that AR^+^ and AR^−^ CARNs had similar proliferation rates as measured by a BrdU incorporation assay during regeneration (Fig. 6a) as well as Ki67 staining at 3 days post pump implantation (Fig. 6g–i; Supplementary Fig. 9b,c; Supplementary Table 5). Instead, we detected fragmented nuclei and positive-cleaved Caspase3 signals in adjacent YFP^+^ cells (Fig. 6j), suggesting that the daughter cells of AR^−^ CARNs were apoptotic. These data demonstrate that CARNs selectively require cell-autonomous AR functions to produce viable luminal cells during prostate regeneration, a unique feature that distinguishes them from average luminal cells in the regressed prostate.

To corroborate the above *in vivo* findings, we also investigated the role of cell-autonomous AR in CARNs using the organoid technique. Lineage-marked CARNs were flow-sorted from castrated and induced Lum^YFP,AR−^ mice based on YFP fluorescence (Fig. 6a,k). Cytospin analysis of the sorted cells showed that 65.8% of them had AR deletion (*n*=356/541, Fig. 6k). Since CARNs are rare, 867 sorted cells were seeded in a well and organoid culture was performed using a standard serum-free protocol^36^. Ten days later, we found nine organoids that were homogeneously YFP positive (Fig. 6l). IF staining revealed that most cells in these organoids were CK18^+^ luminal (Fig. 6m). Importantly, nuclear AR expression was present in all the cells in eight organoids (Fig. 6m), suggesting they were derived from wild-type CARNs. On the basis of these numbers, we calculated the organoid formation efficiency from wild-type CARNs to be 2.7%, comparable to a previous study using a protocol containing serum^35^. The other organoid contained a mixture of AR^+^ and AR^−^ cells (Fig. 6n), indicating its origin from a doublet composed of one AR^+^ and one AR^−^ CARN cell. In a biological repeat experiment, we again did not observe any pure AR^−^ organoid formation. These data demonstrate that AR is also required for CARN stem cell activities in organoid culture.

### Pten loss overrides AR loss in both basal and luminal layers

**Figure 7:**
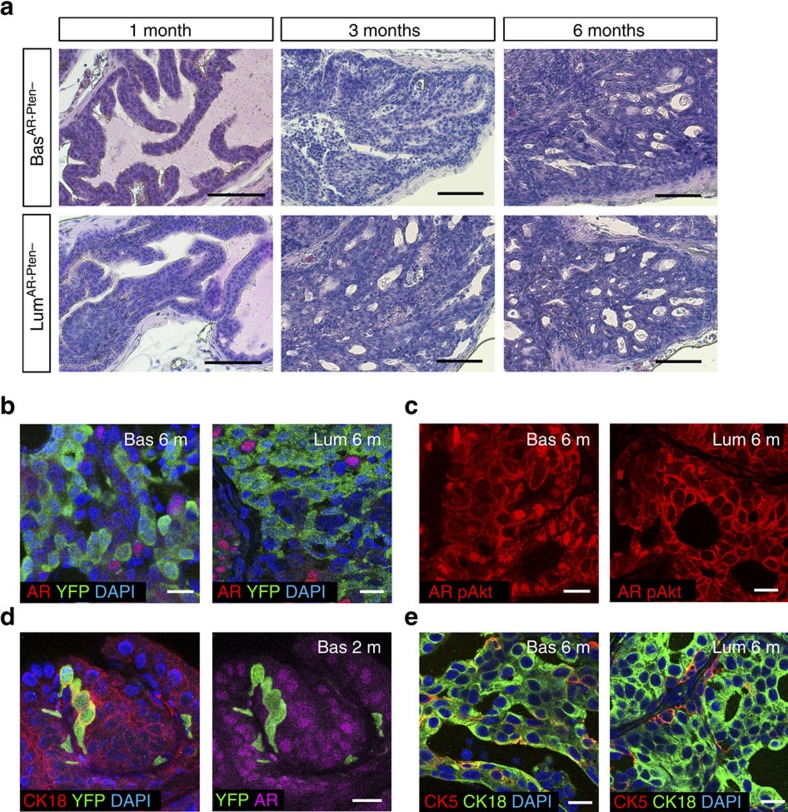
Pten loss overrides AR-loss effects in both basal and luminal cells during cancer initiation. (**a**) H&E staining of Bas^AR−Pten−^ and Lum^AR−Pten−^ AP at 1, 3 and 6 months post induction showing progression to high-grade PIN in both tumours and faster progression in Lum^AR−Pten−^ mice. (**b**,**c**) IF staining showing general absence of AR (nuclear red), enhanced phosphor-Akt (membrane red) in PIN cells and similar tissue morphology in both basal- and luminal-origin tumours at 6 months post induction. (**d**) Representative image of CK18, YFP and AR triple staining showing AR^−^Pten^−^ basal cells differentiate into luminal cells at 2 months post induction. (**e**) IF staining showing basal- and luminal-origin tumours at 6 months post induction primarily show luminal cell features with some cells being intermediate (CK5^+^CK18^+^). Scale bars in **a** correspond to 100 μm and in **b**–**e** to 20 μm.

Having shown the distinct roles of AR in different epithelial cell types in prostate homeostasis, we then explored its cell-type-specific function during cancer initiation. It was discovered that when tumour suppressor gene *Pten* is deleted, both basal and luminal cells can serve as cells of origin for prostate cancer^19^,^20^,^27^. We therefore tested whether AR loss affects tumour initiation from basal and luminal cells under this condition. *CK5-CreER*^*T2*^*; AR*^*flox/Y*^*; Pten*^*flox/flox*^*; R26R-YFP/+* (denoted Bas^AR−Pten−^) mice and *Nkx3.1*^*CreERT2/+*^*; AR*^*flox/Y*^*; Pten*^*flox/flox*^*; R26R-YFP/+* (denoted Lum^AR−Pten−^) mice were induced at 2 month of age and their prostates were analysed at later time points (Supplementary Fig. 10a). One month after induction, the Bas^AR−Pten−^ prostate had an overall normal histology with occasional small foci of hyperplasia. Soon after, Grade II prostatic intraepithelial neoplasia (PIN) lesions began to emerge and were frequent by 3 months after induction. At 6 months after induction, the Bas^AR−Pten−^ prostate contained overwhelmingly Grade IV PINs with cribriform pattern (Fig. 7a; Supplementary Fig. 10b). In comparison, we found that Lum^AR−Pten−^ tumours progressed much faster than Bas^AR−Pten−^ tumours, although their eventual high grade PINs were histologically indistinguishable (Fig. 7a; Supplementary Fig. 10b). These findings are highly analogous to the previous findings about basal- and luminal-origin tumours of *Pten* deletion alone^19^,^20^. IF staining revealed that most cells in 6-month Bas^AR−Pten−^ and Lum^AR−Pten−^ tumours were AR^−^ and phosphor-Akt^+^ (Fig. 7b,c), confirming rapid expansion of *AR*^−^*Pten*^−^ cells. Interestingly, *AR*^−^*Pten*^−^ basal cells behaved like *Pten*^−^ basal cells^19^,^20^ but not *AR*^−^ basal cells, as they readily differentiated into luminal-like cells with enhanced CK18 expression (Fig. 7d). As a result, both Bas^AR−Pten−^ and Lum^AR−Pten−^ tumours were characterized by luminal phenotypes with some cells showing CK5^+^CK18^+^ intermediate features (Fig. 7e). These results suggest that *Pten* deletion plays a dominant role in the *AR*^−^*Pten*^−^ double knockout tumours and can override AR-loss effects in both basal and luminal compartments.

Finally, we tested whether AR^−^ CARNs can serve as the cell of origin for prostate cancer. Lum^AR−Pten−^ mice were first castrated, and then induced and re-administered with androgen (Supplementary Fig. 11a). YFP^+^AR^−^ tumour cell clusters were readily detected in the regenerated prostate (Supplementary Fig. 11b). The PIN lesions looked similar to previously reported Pten^−^ CARN tumours^23^ (Supplementary Fig. 11d), as they expressed high levels of luminal marker CK18 and phosphor-Akt (Supplementary Fig. 11b,c). Therefore, *Pten* deletion can also override the requirement of AR in CARNs to transform these stem cells.

## Discussion

**Figure 8:**
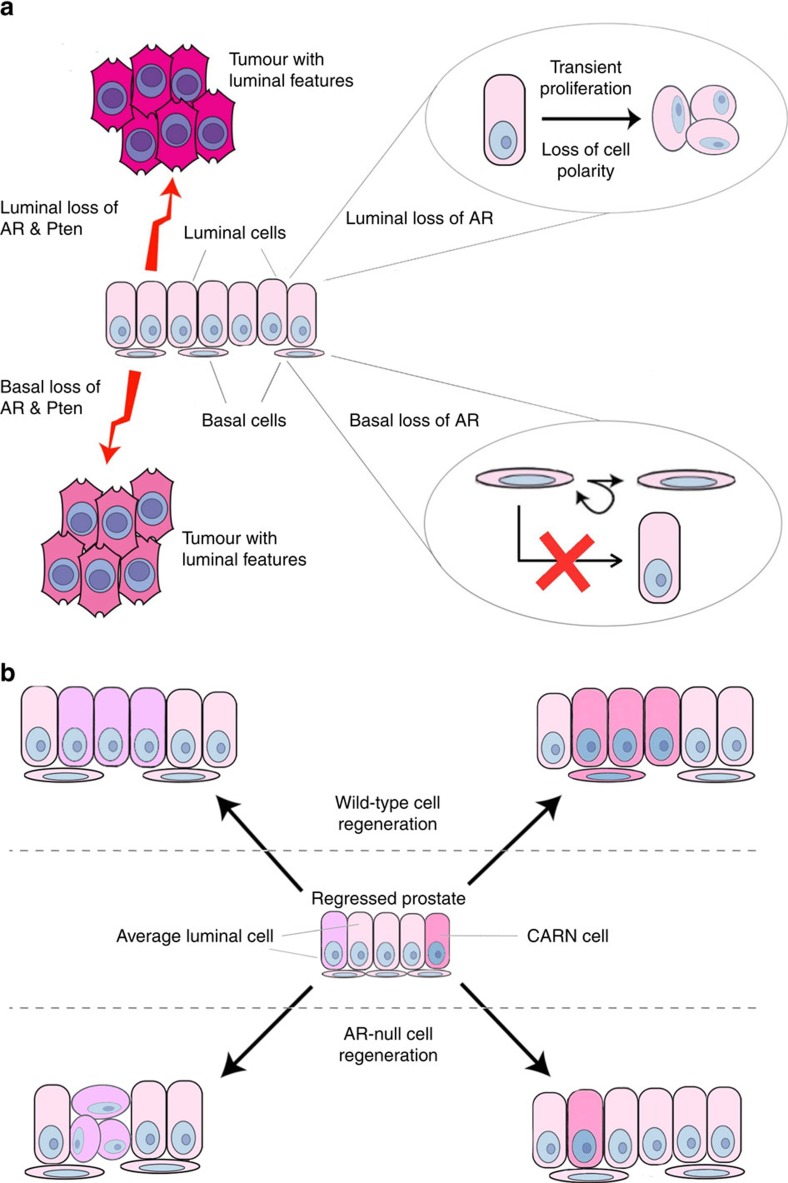
Model of epithelial AR function in the adult prostate. (**a**) In adult prostate homeostasis, cell-autonomous AR is essential for maintaining luminal cell morphology and for basal cells to undergo luminal differentiation. It is not required for androgen-mediated cell survival in either compartment. AR loss in luminal cells also induces a transient proliferation, resulting in a cluster of compact cells that share intermediate cell features. Cell-autonomous AR is not required in either basal or luminal cells for Pten-loss-induced cancer initiation. Despite a faster progression rate of luminal-origin tumours with AR Pten double deletion, both basal- and luminal-origin tumours eventually show luminal cell features. (**b**) In androgen-mediated prostate regeneration, AR is selectively required in luminal stem cell CARNs in order for them to produce viable daughter cells. In contrast, cell-autonomous AR is not required for average luminal cell to proliferate and regenerate upon androgen re-administration, although it ensures the normal morphology and polarity of regenerated luminal cells.

A plethora of studies utilizing both tissue recombination and conditional knockout approaches have established the pivotal role of stromal cell AR in instructing epithelial cell proliferation and differentiation in prostate development^4^,^5^,^6^,^7^,^8^,^9^,^10^. Here, we demonstrate that AR in adult prostate epithelial cells plays diverse roles in maintaining normal tissue structure, and is crucial for the differentiation capability of adult prostate stem cells in both basal and luminal layers *in vivo* (Fig. 8).

The present study differs from several previous studies of AR conditional knockout in the prostate. Using *Probasin-Cre* (*Pb-Cre*) lines to delete AR in the developing epithelium, three studies reported increased basal cell proliferation, but conflicting findings regarding luminal cell behaviours. These luminal phenotypes included formation of cell clusters and higher proliferation^14^, little apoptosis in the epithelium, but sloughing of luminal cells into the lumen^15^ and high levels of luminal apoptosis and lower proliferation^16^. The cause for these discrepancies is unknown. In our AR deletion experiments, we did not observe basal cell over-proliferation or luminal cell apoptosis/anoikis. One key distinction is that *Pb-Cre* lines become active in early postnatal development^41^, whereas the inducible basal and luminal CreER lines were activated by tamoxifen at the adult stage. Therefore, our data reflect homeostatic events in the mature organ, whereas previous studies likely captured developmental consequences of AR loss. As demonstrated previously for prostate basal cells, and stem cells in other organs such as the mammary gland^19^,^42^,^43^, cell behaviours including plasticity can be drastically different between developmental stage and adulthood. During postnatal development, when basal and intermediate cells are actively producing luminal cells^18^, AR loss in luminal cells at this stage might activate a compensatory mechanism to stimulate basal cell proliferation. Alternatively, AR may cell-autonomously repress proliferation of postnatal basal cells, as one study found higher basal cell proliferation when AR was deleted at an early stage by *CK5-Cre*^17^. Such repression by AR may no longer be needed in the adult basal cells, given that the mature prostate is relatively quiescent and that the plasticity of adult basal cells has become restricted.

Similarly, different timing of Cre activation may also contribute to the reported discrepancies of AR-loss effects on luminal cells. Moreover, characterization of the *Pb-Cre4* line has determined that its expression is not only in the epithelium, but also in stromal cells (ref. 41; and our unpublished observations). Therefore, the reported luminal apoptosis phenotypes may alternatively be attributed to *Pb-Cre4* leakage and a decrease in stromal AR, since AR conditional knockout in the prostate mesenchyme led to higher epithelial cell apoptosis^9^,^10^. Our data showing the dispensability of luminal cell-autonomous AR in their survival and regeneration further support the idea that androgen regulates luminal cell behaviours primarily in a non-cell-autonomous fashion. Instead, luminal cell-autonomous AR is important for maintaining normal luminal cell morphology, possibly through regulating cytoskeleton, cell adhesion and TGF-β signalling pathways, as indicated by our transcriptome analyses. One potential caveat of the *Nkx3.1*^*CreERT2*^ line is that the Cre knock-in disrupts the *Nkx3.1* gene, which orchestrates a transcriptional regulatory network important for prostate cell fate^44^. However, the *Nkx3.1-CreER*^*T2/+*^ mice that we were using were heterozygous and still expressed Nkx3.1 in luminal cells (Supplementary Figs 4d and 6c), and *Nkx3.1-/+* mice were shown to only have very mild phenotypes at old age^45^. Besides, without AR, *Nkx3.1* expression is down-regulated anyway. Therefore, we think this technicality is unlikely to affect our conclusions, although the possibility that *nkx3.1* heterozygosity somehow further altered AR^−^ luminal cell properties cannot be totally ruled out.

Interestingly, we find cell-autonomous AR to be essential for stem cell functions in both basal and luminal compartments, although the underlying mechanisms appear to be different. Androgen has been shown to promote basal to luminal cell differentiation in prostate spheres *in vitro*^37^,^46^ and in organogenesis^47^. Our data for the first time demonstrate the requirement of cell-autonomous AR in adult basal cells for their luminal differentiation *in vivo*. We speculate that rare adult BSCs preserve such a mechanism from postnatal basal cells so that they may step in to generate luminal cells in response to environmental cues such as injury^48^,^49^. The discovery of a selective AR requirement in luminal CARNs is intriguing. In the regressed prostate, CARNs can regenerate luminal cells (and basal cells to a lesser extent) upon androgen re-administration^23^. Later studies showed that, average regressed luminal cells, which are *Nkx3.1*-low/negative, also proliferate^19^ and contribute to luminal cell regeneration^20^,^22^. This raises the question as to whether CARNs are a functionally unique population or rare luminal cells that happen to retain the expression of *Nkx3.1*. Our data lend support to the former. We show that CARNs, unlike average luminal cells, are sensitive to cell-autonomous AR levels. Importantly, the survival and proliferation of AR^−^ CARNs are not affected, but their differentiated daughter cells are apoptotic. Programed cell death upon cell division was best illustrated in *C. elegans* neuroblast development, where asymmetric cell division usually generates a smaller daughter cell fated to die, through mechanisms involving EGL-1 and Snail-related proteins^50^,^51^. It is tempting to speculate that AR loss in CARNs may activate similar mechanisms. Future research will shed light on this, but our organoid culture data and the absence of apoptotic signal in Bas^YFP,AR−^ tissues suggest that AR is directly involved in BSC differentiation while its role in CARNs is relatively indirect.

Whether AR is tumour-suppressing or -promoting in prostate cancer is under heated debate^11^,^12^,^52^, and the answer is likely dependent on the specific cell type and progression stage. We found that, despite a transient (1–2 weeks) over-proliferation of AR^−^ luminal cells, their proliferation rates decreased back to normal thereafter, and AR^−^ cell clusters never expanded to colonize the tissue (Fig. 3), suggesting they were not tumorigenic. These data, which were obtained in adult animals, challenge the previous notion that epithelial AR plays a tumour suppressor role during early cancer initiation, since previous experiments were performed at the postnatal stage^15^,^52^. We acknowledge that in our experiments AR was deleted in ∼20% of all luminal cells. While this number is comparable to luminal marking efficiency in a previous study^18^, it is conceivable that the rest AR^+^ luminal cells can signal to AR^−^ ones and influence their behaviours. Should the AR deletion efficiency be higher, we might observe more prominent PIN-like clustered-cell phenotypes or even un-checked clonal growth. However, we think the later scenario is unlikely, since we did not notice difference with respect to individual cell morphology or proliferation rate between small and large AR^−^ luminal cell clusters. In future investigations, it will be important to determine whether paracrine signals from AR^+^ luminal cells or perhaps basal and stromal cells caused the over-proliferation of AR^−^ luminal cells to be transient, and whether cell competition exists between AR^−^ and AR^+^ populations. In any case, our luminal AR deletion context may be more physiologically relevant than a situation where deletion occurs ubiquitously (for example, using *Pb-Cre*), since the appearance of any AR^−^ luminal cells in human prostate should start from a small scale. Finally, our data of *AR Pten* double knockout in adult basal and luminal cells are consistent with a previous study showing the dispensability of epithelial AR in *Pten*-null tumour initiation^28^. Mechanistically, Pten loss was found to suppress AR transcriptional output^28^, thereby probably rendering cell-autonomous AR loss to be partially redundant. Indeed, while *Pten* undergoes copy number loss as a relatively early event in human prostate carcinogenesis^53^,^54^, mutations of the AR gene have recently been found exclusively in metastatic, castration-resistant human prostate cancer^55^, indicating that AR plays a more prominent role in later-stage cancer progression.

## Methods

### Mouse strains and genotyping

The *Nkx3.1*^*CreERT2/+*^ targeted allele^23^, *CK5-CreER*^*T2*^ transgenic line^29^, *AR*^*flox*^ allele^31^, *Pten*^*flox*^ allele^56^, *R26R-CAG-YFP* line^30^ and *R26R-YFP* line^57^ were described previously. Animals were maintained in C57BL/6N background. Genotyping was performed by PCR using tail genomic DNA, with the following primer sequences: *Nkx3.1* wild-type allele, 5′-CTCCGCTACCCTAAGCATCC-3′ and 5′-GACACTGTCATATTACTTGGACC-3′; *CreER*^*T2*^ allele, 5′-CAGATGGCGCGGCAACACC-3′ and 5′-GCGCGGTCTGGCAGTAAAAAC-3′; *AR*^*flox*^ allele, 5′-GTTGATACCTTAACCTCTGC-3′ and 5′-CTTCAGCGGCTCTTTTGAAG-3′; *Pten*^*flox*^ allele, 5′-ACTCAAGGCAGGGATGAGC-3′ and 5′-GTCATCTTCACTTAGCCATTGG-3′; *R26R-YFP* allele, 5′-GCGAAGAGTTTGTCCTCAACC-3′ (mutated forward), 5′-GGAGCGGGAGAAATGGATATG-3′ (wild-type forward) and 5′-AAAGTCGCTCTGAGTTGTTAT-3′ (wild-type and mutated reverse); *R26R-CAG-YFP* allele, 5′-AAGGGAGCTGCAGTGGAGTA-3′ (wild-type forward), 5′-CCGAAAATCTGTGGGAAGTC-3′ (wild-type reverse), 5′-ACATGGTCCTGCTGGAGTTC-3′ (mutated forward), 5′-GGCATTAAGCAGCGTATCC-3′ (mutated reverse).

### Mouse procedures and surgery

For tamoxifen induction, mice were administered 9 mg per 40 g body weight tamoxifen (Sigma) suspended in corn oil by oral gavage once daily for 4 consecutive days. Castration of adult male mice was performed using standard techniques, with the fully regressed state attained at 4 weeks after castration. For prostate regeneration, testosterone (Sigma) was dissolved at 25 mg ml^−1^ in 100% ethanol and diluted in PEG-400 to a final concentration of 7.5 mg ml^−1^. Testosterone was administered for 4 weeks at a rate of 1.875 μg h^−1^ delivered by subcutaneous implantation of mini-osmotic pumps (Alzet), which yields physiological levels of serum testosterone^58^. All animal experiments received approval from the Institutional Animal Care and Use Committee at UCSC. No statistical method was used to predetermine mouse sample size. The mouse experiments were not randomized.

### BrdU incorporation assay

BrdU (Sigma) was dissolved in PBS (10 mg ml^−1^) and administered by intraperitoneal injection twice daily (0.1 ml per dose) for 7 or 12 consecutive days during homeostasis or regeneration to label proliferating cells.

### Tissue collection and flow cytometry

For histological and IF analyses, individual prostate lobes were dissected and fixed in 4% paraformaldehyde for subsequent cryo-embedding in OCT compound (Sakura), or fixed in 10% formalin followed by paraffin embedding.

For flow cytometry, prostate tissues were dissected and minced to small clumps, followed by enzymatic dissociation with 0.2% collagenase I (Invitrogen) in DMEM media with 5% FBS for 3 h at 37 °C. Tissues were digested with 0.25% Trypsin-EDTA (StemCell Technologies) for 1 h at 4 °C, passed through 21- to 26-gauge syringes and filtered through a 40-μm cell strainer to obtain single-cell suspensions. Dissociated prostate cells were suspended in Hanks' Balanced Salt Solution Modified/2% FBS. ROCK inhibitor Y-27632 (StemCell Technologies) was added at 10 uM throughout the whole process to inhibit luminal cell death. Dead cells were excluded by propidium iodide staining and cell sorting was performed on a BD FACS Aria II instrument in the Flow Cytometry Shared Facility of UCSC. Antibodies used for sorting luminal and basal cells are listed in Supplementary Table 6.

### Prostate organoid culture

Flow-sorted YFP^+^ basal or CARN cells were washed with advanced DMEM/F12 (Life Technologies), and resuspended in 10 μl advanced DMEM/F12 and 30 μl Matrigel per well in the Nunc Lab-Tek II CC2 Chamber Slide System (Fisher). Chamber slide was put upside down in the 37 °C cell culture incubator for 15 min to let the matrigel solidify. Mouse prostate organoid culture medium was prepared according a previous protocol^36^. Briefly, the following components were added to advanced DMEM/F12 medium, B27 (50 × diluted), HEPES 1 M (100 × diluted), GlutaMAX (100 × diluted), Penicillin-streptomycin (100 × diluted), N-acetylcysteine (1.25 mM), EGF (50 ng ml^−1^), A83-01 (200 nM), Noggin (100 ng ml^−1^), R-spondin 1 (500 ng ml^−1^), DHT (1 nM), Y-27632 dihydrochloride (10 μM). Organoid culture medium was prewarmed before adding to the wells. The medium was changed every 2–3 days. Organoids were fixed in 4% PFA for 20 min at room temperature, and collected and resuspended in Histogel. Organoids/Histogel mixture was let to solidify at 4 °C and was embedded in OCT after sucrose treatment. *In situ* organoid images were taken using the Keyence microscope in the Microscopy Shared Facility of UCSC. Organoid sizes were quantified using ImageJ.

### Western blot

Total protein was extracted from flow-sorted cells using T-PER Tissue Protein Extraction Reagent (Fisher), separated by SDS-PAGE and transferred onto PVDF membrane according to standard protocols. Membranes were probed with antibodies directed against AR (sc-815, Santa Cruz Biotechnology, 1:500) and β-actin (sc-47778, Santa Cruz Biotechnology, 1:500). Signal was visualized with secondary HRP conjugated antibodies and Clarity Western ECL Substrate (Biorad). Full size images are presented in Supplementary Fig. 12.

### Quantitative real-time PCR analysis

Quantitative real-time PCR was carried out using Power SYBR Green PCR Master Mix (Life Technology) in the ViiA 7 Real-Time PCR instrument. cDNA samples were diluted 1:100 for all analyses, which were performed in quadruplicate. Expression values were obtained using the ΔΔCT method and normalized to β-actin (Actb) expression; average values are shown as the mean±s.d. Primer sequences are provided in Supplementary Table 7.

### Histology and immunofluorescence staining

H&E staining was performed using standard protocols on 6 μm paraffin sections. Histological assessments were performed using a published classification of mouse PIN lesions^59^. For immunohistochemical staining, 6 μm paraffin sections were deparaffinized in xylene, followed by boiling in antigen unmasking solution (Vector Labs). Slides were blocked in 10% normal goat serum (NGS; Vector Labs), and incubated with primary antibodies diluted in 10% NGS overnight at 4 °C. Secondary antibodies were obtained from Vectastain ABC kits (Vector Labs) and diluted 1:250. Signal was enhanced using the Vectastain ABC system and visualized with the NovaRed Substrate Kit (Vector Labs). Slides were counterstained with Harris modified haematoxylin (1:4 diluted in H_2_O; Fisher Scientific) and mounted with Clearmount (American MasterTech). H&E and immunohistochemical staining was obtained using a Zeiss Axio Imager in the Microscopy Shared Facility of UCSC.

IF staining was performed using 6 μm cryosections (3 μm for staining adjacent sections), which were incubated in 3% H_2_O_2_ and Antigen Unmasking Solution (Vector Labs) for 15 min. Samples were incubated with 10% NGS and primary antibodies diluted in 10% NGS overnight at 4 °C. Samples were then incubated with secondary antibodies (diluted 1:500 in PBST) labelled with Alexa Fluor 488, 555 or 647 (Invitrogen/Molecular Probes). Detection of Nkx3.1 was enhanced using tyramide amplification (Invitrogen/Molecular Probes) by incubation of slides with HRP-conjugated secondary antibody (1:100 dilution) (Invitrogen/Molecular Probes), followed by incubation with tyramide 555 for 6 min. Slides were mounted with VectaShield mounting medium with DAPI (Vector Labs). IF staining was imaged using a Leica TCS SP5 spectral confocal microscope in the Microscopy Shared Facility of UCSC. All primary antibodies and dilutions used are listed in Supplementary Table 6.

### Cell counting for lineage analyses and statistics

The investigators were blinded to the ID/genotype of the mice before performing cell counting. Cell numbers were counted manually using confocal × 40 and × 63 photomicrographs across tissue sections. Basal cells were identified based on lack of CK18 staining, positivity for CK5 staining, and/or shape of the cells (oval or triangular) and their positions at the basement of the epithelium. Luminal cells were determined based on positive CK18 staining and/or shape of the cells (columnar) and their positions at the apical side of the epithelium. Statistical analyses were performed using a two-sided student's *t*-test, Fisher's exact test, or *χ*^2^ test as appropriate. At least three animals for each experiment or genotype were analysed. The variances were similar between the groups that were being statistically compared.

### RNA sequencing

Total RNA from FACS-purified luminal cells was isolated using the RNeasy Micro Kit (Qiagen). RNA in each sample was reverse transcribed and amplified into cDNA using the Ovation RNA-Seq System V2 kit (Nugen). The quantity and quality of each sample was measured using an Agilent 2100 Bioanalyzer. Samples were sent to the Columbia Genome Center for library construction and sequencing. The single-end sequencing was performed on the Illumina HiSeq 2000 platform. bc and bcl2fastq (v1.8.4) was used for converting BCL to fastq format, coupled with adaptor trimming. Sequencing reads were then mapped to mouse genome (mm.9) using TopHat (v2.0.4) by allowing up to four mismatches and ten maximum multiple hits. Expression of genes in the RNA-seq data was measured by calculating reads per kilobase per million mapped reads (FPKM value) using cufflinks (v2.0.2) software with default settings.

### Principal components analysis and clustering analysis

PCA was performed on scaled data, where the data value was adjusted by subtracting its mean across all samples and dividing by its s.d., z=(x-mean)/s.d. For decreasing the effects of potential outliers, the too highly (highest 100 genes according to average FPKM in all samples) or too lowly expressed genes (FPKM<10 in all samples) were filtered out. ‘pccomp' command in R v3.2.2 was used for PCA analysis. The gene hierarchical clustering was done by using open source clustering software^60^. Here, the Pearson correlation distance was calculated and the average linkage clustering algorithm was chosen.

### Gene expression and pathway analyses

Differential expression was estimated using the empirical Bayes methods (limma package^61^ v3.24.15 in R v3.2.2). Fold-change analysis was performed on data regenerated by reverse log transformation. The differentially expressed genes (FDR <0.1, and fold change >2) were extracted and fed to the DAVID website^40^,^62^ for the enriched pathway analysis.

### Data availability

The data that support the findings of this study are available from the corresponding author on request. RNA-seq expression data are deposited in the Gene Expression Omnibus database under GSE76724.

## Additional information

**How to cite this article:** Xie, Q. *et al*. Dissecting cell-type-specific roles of androgen receptor in prostate homeostasis and regeneration through lineage tracing. *Nat. Commun.* **8,** 14284 doi: 10.1038/ncomms14284 (2017).

**Publisher's note:** Springer Nature remains neutral with regard to jurisdictional claims in published maps and institutional affiliations.

## Supplementary Material

Supplementary Information Supplementary Figures and Supplementary Tables

Supplementary Data 1 List of genes up-regulated in AR- luminal cells.

Supplementary Data 2 List of genes down-regulated in AR- luminal cells.

Supplementary Data 3 List of enriched molecular pathways in AR- luminal cells by DAVID GO analysis.
