# Supplementary material for: Dissecting cell-type-specific roles of androgen receptor in prostate homeostasis and regeneration through lineage tracing
Source: Nat Commun. 2017 Jan 23;8:14284. doi: 10.1038/ncomms14284 (PMC5264212; doi:10.1038/ncomms14284)
Supplement: Supplementary Information — Supplementary Figures and Supplementary Tables [file ncomms14284-s1.pdf]

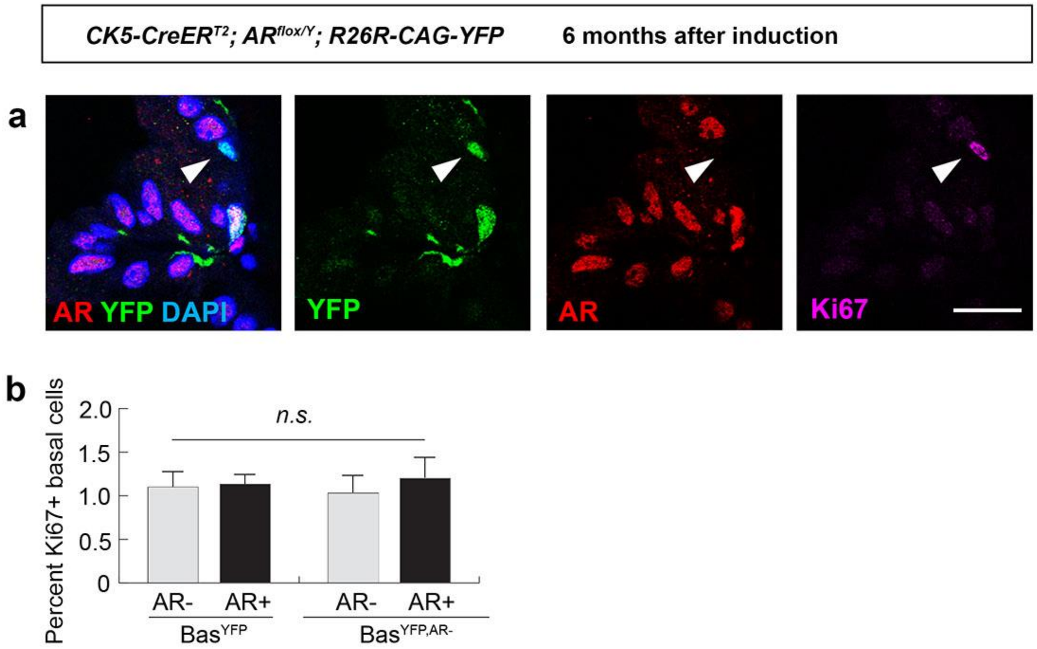

Supplementary Figure 1. Characterization of AR$^+$ and AR$^-$ basal cell proliferation by Ki67 staining.

(**a**) Representative IF staining image showing an AR$^-$ basal cell that is Ki67$^+$ (arrowhead) at 6 months post induction in Bas$^{YFP,AR-}$ mice. (**b**) Quantitation of the percentages of Ki67$^+$ basal cells among AR$^+$ and AR$^-$ basal cells in Bas^{YFP} and Bas$^{YFP,AR-}$ mice 6 months post induction showing that different basal cell populations have the same proliferation rate by t-test. Scale bar corresponds to 20 microns. Error bars correspond to one standard deviation.

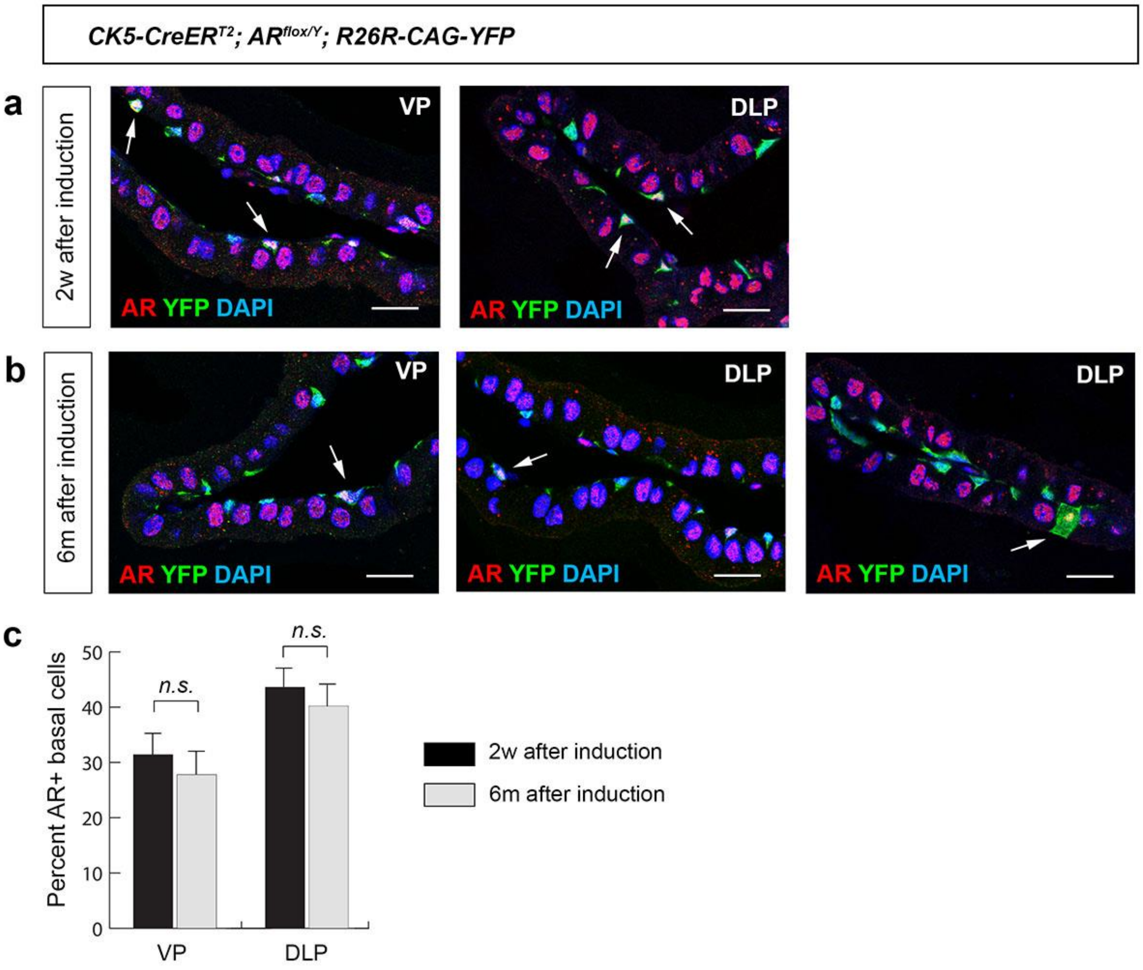

Supplementary Figure 2. Lineage analysis of AR^{+} and AR^{-} basal cells in VP and DLP lobes during prostate homeostasis.

(**a**) Representative IF staining images showing deletion of AR in a subset of adult basal cells in Bas$^{YFP,AR-}$ VP and DLP 2 weeks post induction. Arrows point to marked basal cells that remained AR^{+} (escaped deletion). (**b**) Representative IF staining images of Bas$^{YFP,AR-}$ VP and DLP 6 months post induction showing normal basal cell homeostasis and rare luminal differentiation from AR^{+} basal cells. Arrows point to AR^{+} basal cells in the left and middle panels, and to a rare differentiated AR^{+}YFP^{+} luminal cell in the right panel. (**c**) Quantitation of the percentage of AR^{+} basal cells among total basal cells in Bas$^{YFP,AR-}$ VP and DLP at 2 weeks and 6 months post induction showing that the ratio is constant during homeostasis by t-test. Scale bar corresponds to 20 microns. Error bars correspond to one standard deviation.

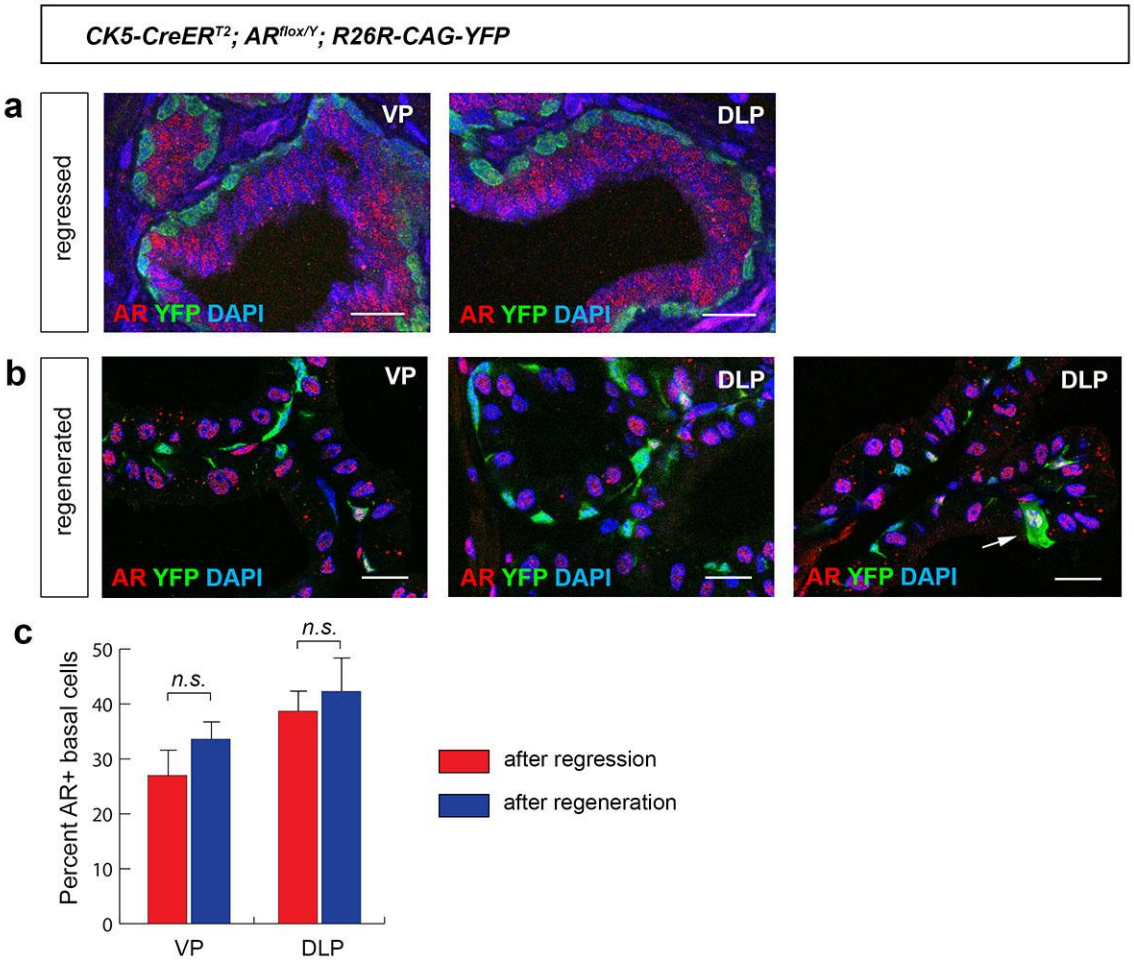

Supplementary Figure 3. Lineage analysis of AR$^+$ and AR$^-$ basal cells in VP and DLP lobes during prostate regression-regeneration.

(**a**) Representative IF staining images showing deletion of AR in a subset of basal cells in the regressed VP and DLP of Bas$^{YFP,AR-}$ mice. (**b**) Representative IF staining images of Bas$^{YFP,AR-}$ VP and DLP after regeneration showing regenerated tissue containing mostly YFP$^+$ basal cells and rare AR$^+$YFP$^+$ luminal cells (arrow in the right panel) derived from AR$^+$ basal cells. (**c**) Quantitation of the percentage of AR$^+$ basal cells among total basal cells in Bas$^{YFP,AR-}$ VP and DLP before and after regeneration, showing that the ratio did not change in this procedure by t-test. Scale bar corresponds to 20 microns. Error bars correspond to one standard deviation.

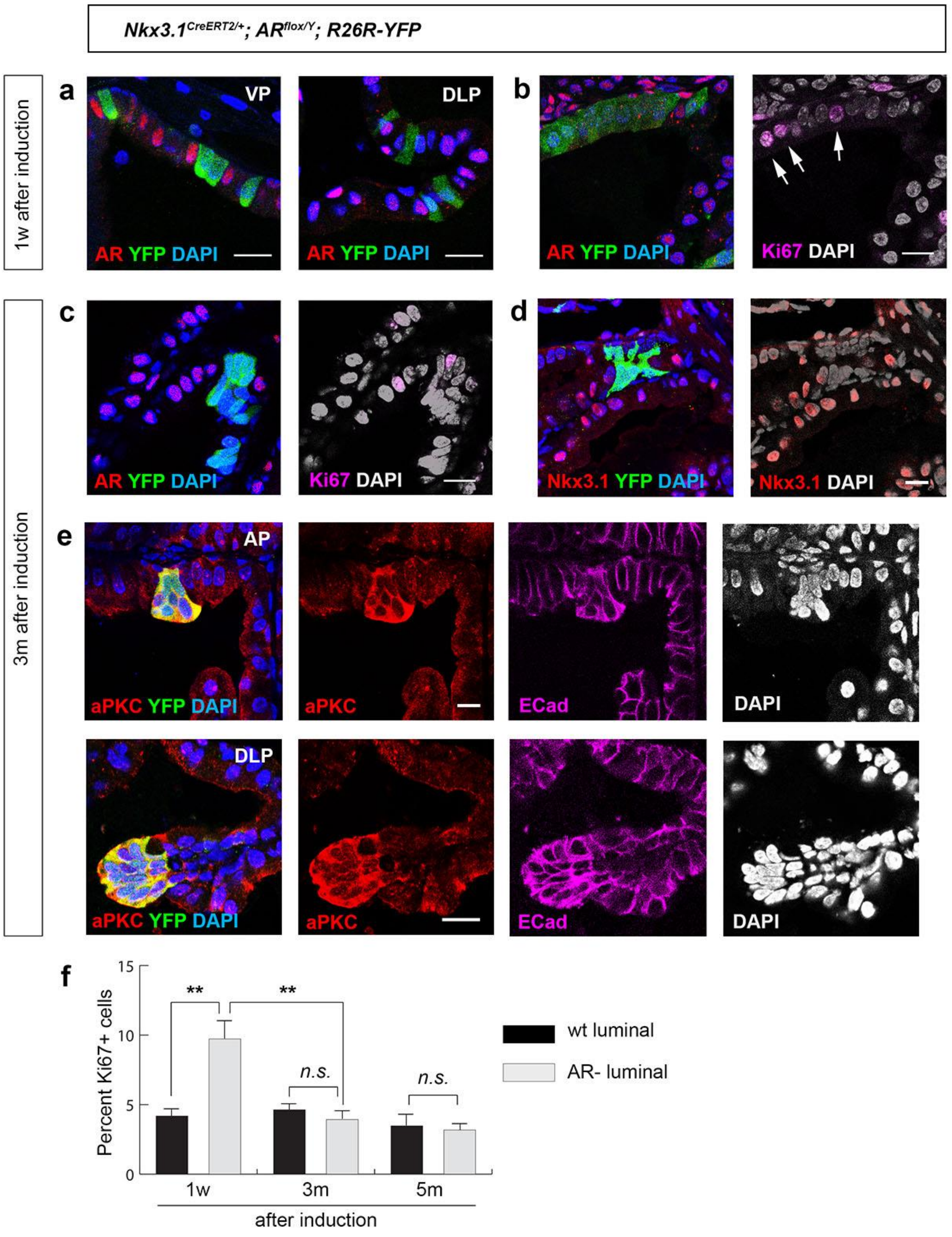

Supplementary Figure 4. Phenotypic characterization of AR$^-$ luminal cells.

(**a**) IF staining showing simultaneous deletion of AR and marking by YFP in a subset of adult luminal cells in the VP and DLP of Lum$^{YFP,AR-}$ mice 1 week post induction. (**b**,**c**) Representative Ki67 staining images showing that many AR$^-$ luminal cells were proliferating (arrows) 1 week post induction (**b**) and relatively few were proliferating 3 months post induction (**c**). (**d**) IF staining showing *Nkx3.1* expression was down-regulated in AR$^-$ luminal cells. (**e**) IF staining showing enhanced and mislocalized αPKC and E-Cadherin expression on all sides of AR$^-$ luminal cells 3 months post induction. (**f**) Quantitation of wild-type and AR$^-$ luminal cell proliferation rate by Ki67 staining at different analysis time points in prostate homeostasis of Lum$^{YFP,AR-}$ mice showing a transient over-proliferation in AR$^-$ luminal cells at 1 week post induction. ** $p<0.001$, n.s., by t-test. Scale bars correspond to 20 microns. Error bars correspond to one standard deviation.

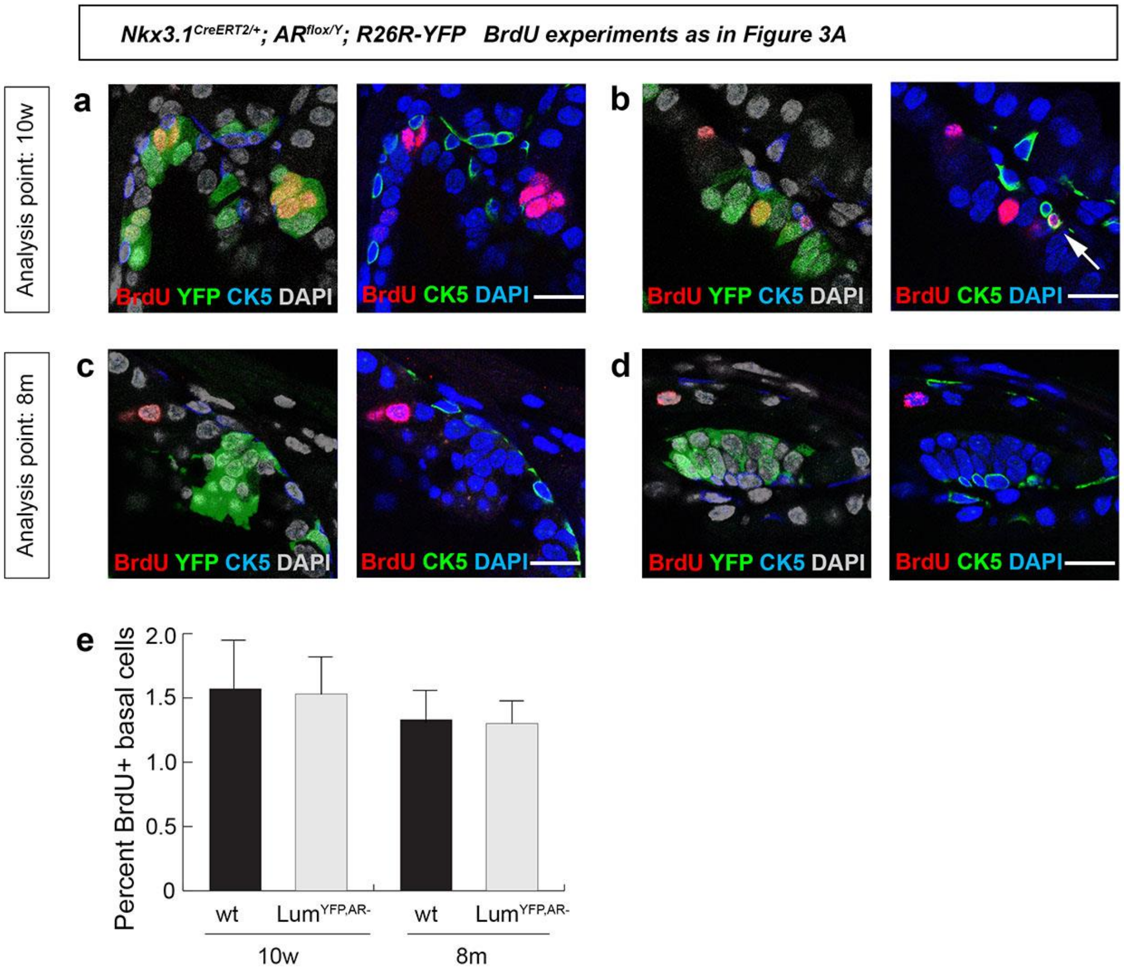

Supplementary Figure 5. Basal cell proliferation is normal in Lum$^{YFP,AR-}$ mice.

(**a**,**b**) IF staining of CK5, BrdU, and YFP in a BrdU incorporation assay in Lum$^{YFP,AR-}$ mice analyzed at 10 weeks of age (2 weeks post induction) showing many AR$^-$ luminal cells were proliferating while basal cell proliferation remained slow. Arrow points to a BrdU$^+$ basal cell. (**c**,**d**) IF staining of CK5, BrdU, and YFP in a BrdU incorporation assay in Lum$^{YFP,AR-}$ mice analyzed at 8 months of age (6 months post induction) showing slow proliferation in both AR$^-$ luminal cells and basal cells. (**e**) Quantitation of the percentages of BrdU$^+$ basal cells in the BrdU incorporation assays analyzed at 10 weeks and 8 months of age showing that basal cell proliferation rates in wild-type mice and Lum$^{YFP,AR-}$ mice are similar at both time points by t-test. Scale bars correspond to 20 microns. Error bars correspond to one standard deviation.

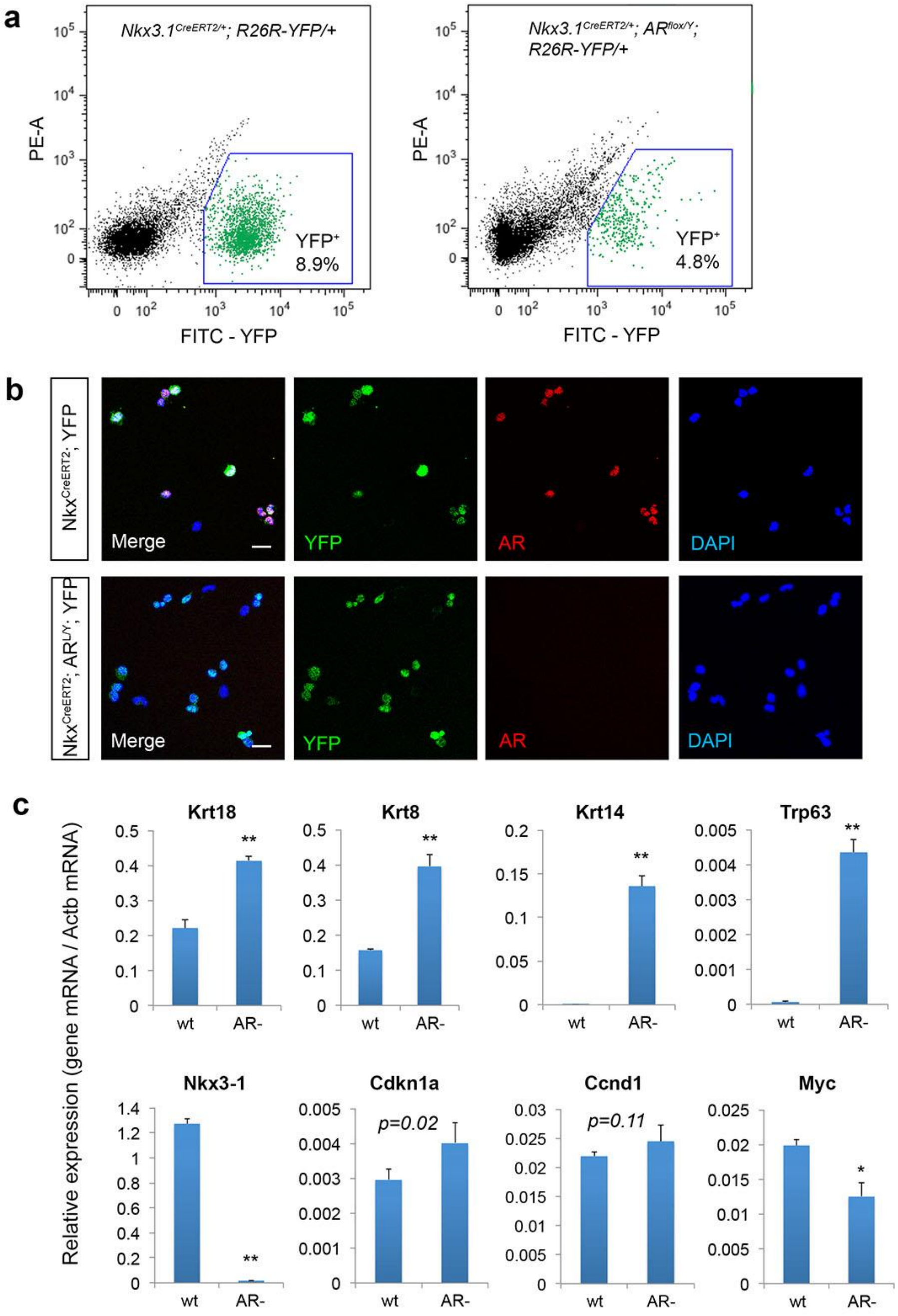

Supplementary Figure 6. Isolation and molecular comparison of wild-type and AR^{-} luminal cells.

(**a**) FACS plot showing the gate drawn for sorting wild-type luminal cells from Lum^{YFP} mice (left) and AR^{-} luminal cells from Lum$^{YFP,AR-}$ mice (right) based on YFP fluorescence. (**b**) IF staining of cytospin preparations confirming co-localization of YFP and AR in sorted wild-type luminal cells and absence of AR in sorted AR^{-} luminal cells. Scale bars correspond to 20 microns. (**c**) Quantitative real-time PCR analysis of selected genes in sorted wild-type and AR^{-} luminal cells. Gene expression levels were normalized to β-actin expression. *Krt5* expression was not detected in either cell populations. * $p<0.01$, ** $p<0.001$ by t-test. Error bars correspond to one standard deviation.

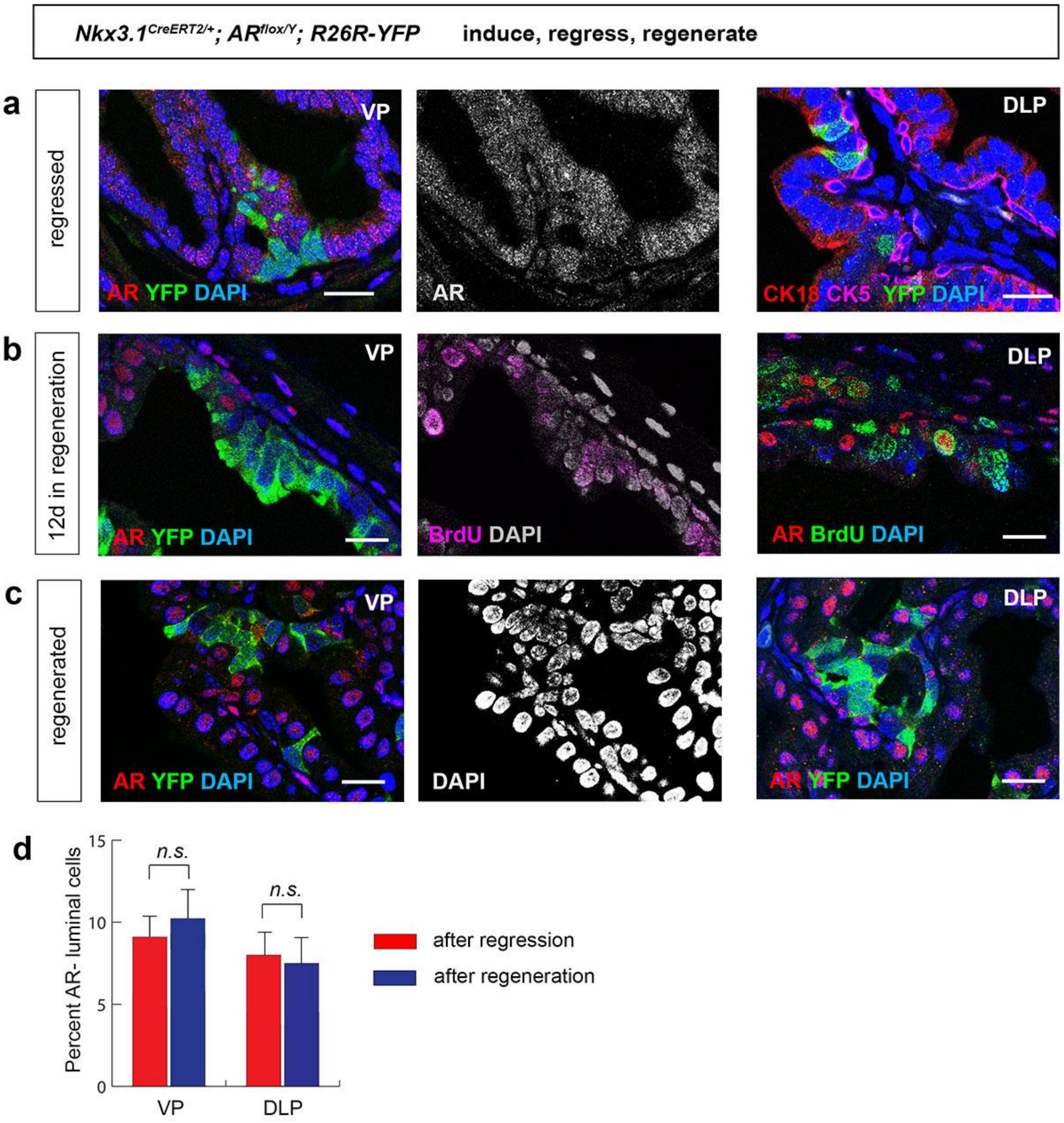

Supplementary Figure 7. Cell-autonomous AR is dispensable for luminal cell regeneration in the VP and DLP lobes.

(**a**) Representative IF staining image showing that AR^- luminal cells were present in regressed VP and DLP, were marked by YFP, and were $CK18^+CK5^-$. (**b**) IF staining in a 12-day BrdU incorporation assay during prostate regeneration, showing many AR^- luminal cells in the VP and DLP were proliferating. (**c**) Representative IF staining image showing the presence of AR^-YFP^+ cell clusters in the VP and DLP after regeneration. (**d**) Quantitation of the percentages of AR^- luminal cells among total luminal cells during the course of prostate regression-regeneration showing the ratios remained constant in the VP and DLP by t-test. Scale bars correspond to 20 microns. Error bars correspond to one standard deviation.

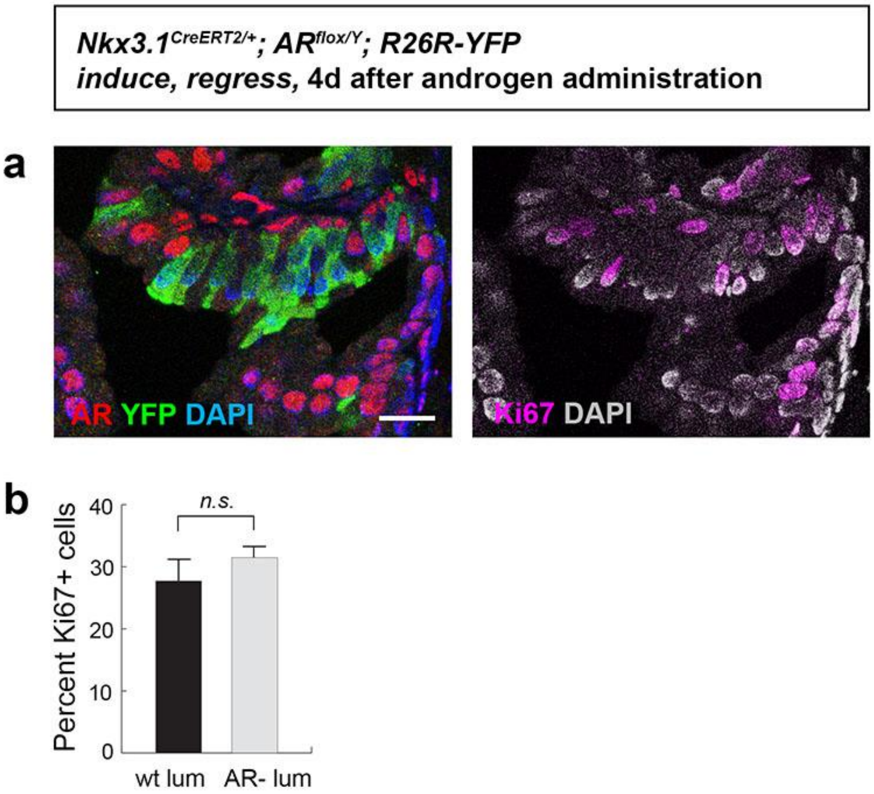

Supplementary Figure 8. Characterization of wild-type and AR$^-$ luminal cell proliferation in regeneration by Ki67 staining.

(**a**) Representative IF staining showing the presence of many Ki67$^+$ cells in both wild-type luminal cells and AR$^-$ luminal cells at 4 days after androgen re-administration. (**b**) Quantitation of the percentages of Ki67$^+$ luminal cells at 4 days after androgen re-administration showing no difference by t-test in the proliferation rate of wild-type and AR$^-$ luminal cells. Scale bars correspond to 20 microns. Error bars correspond to one standard deviation.

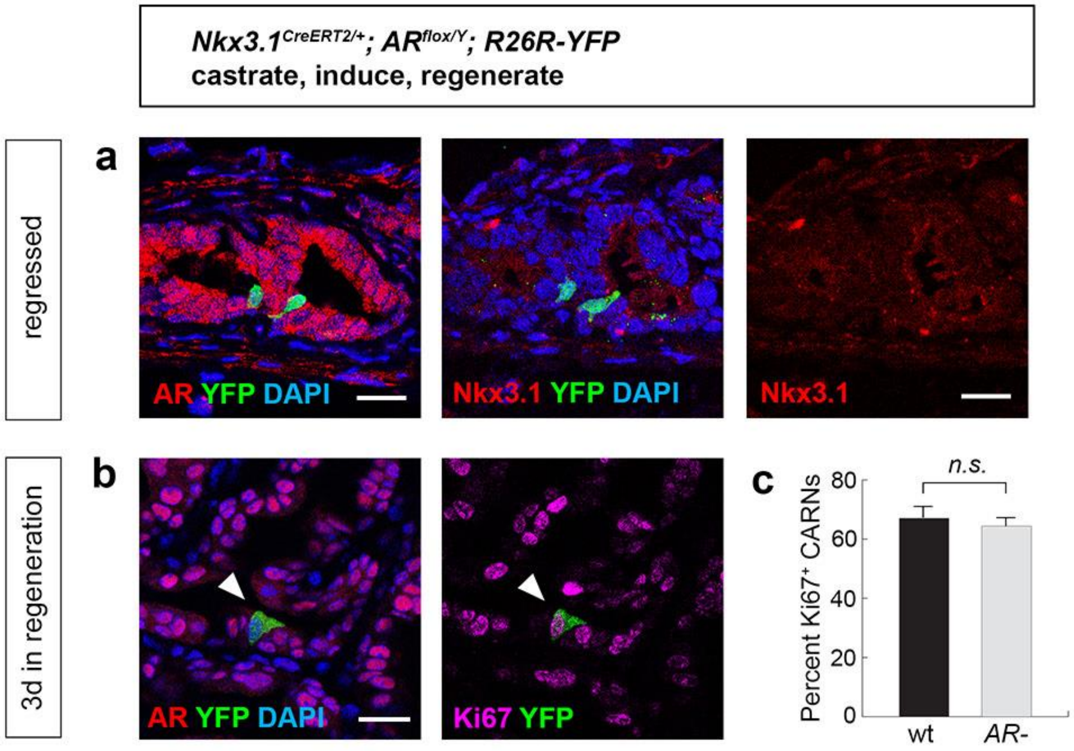

Supplementary Figure 9. Characterization of AR$^-$ CARNs in the regressed prostate and in regeneration by Ki67 staining.

(**a**) IF staining of adjacent sections (left vs. middle and right panels) showing that AR$^-$ CARNs in the regressed prostate (marked by YFP) were Nkx3.1-negative. (**b**) Representative image of Ki67, AR, YFP triple staining showing an AR$^-$ CARN (arrowhead) was proliferating at 3 days after androgen re-administration. (**c**) Quantitation of the percentages of Ki67$^+$ CARNs at 3 days after androgen re-administration showing no difference by t-test between wild-type CARNs and AR$^-$ CARNs. Scale bars correspond to 20 microns. Error bars correspond to one standard deviation.

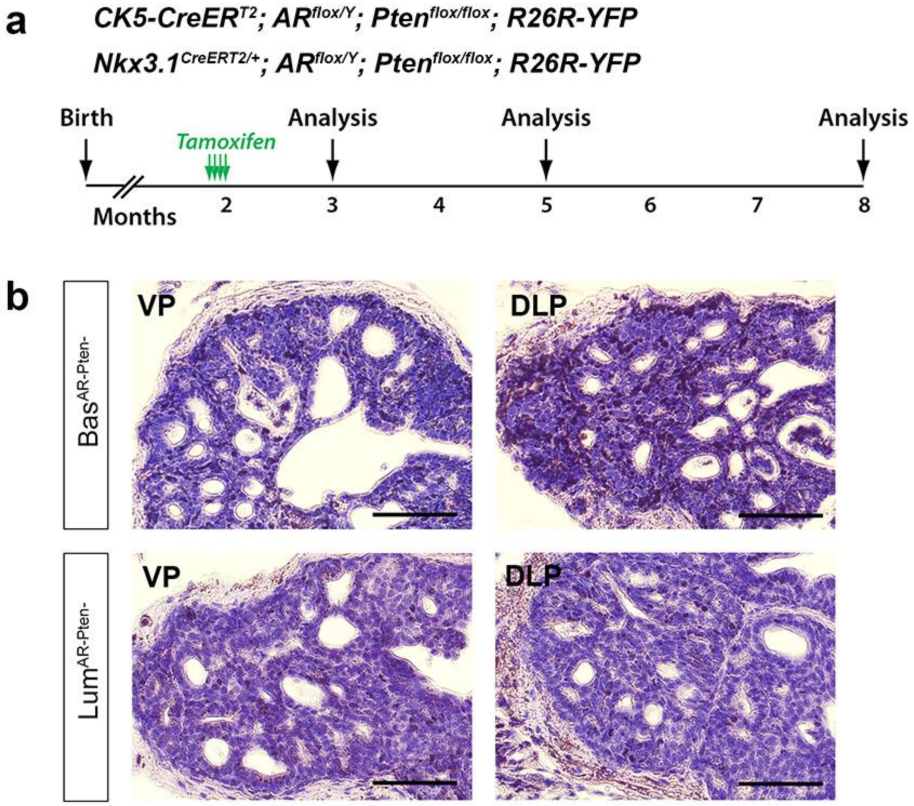

Supplementary Figure 10. Basal- and luminal-origin tumors of AR Pten double knockout in VP and DLP have similar histology.

(**a**) Strategy for inducing basal- and luminal-origin tumors with AR and Pten double deletion. (**b**) H&E staining showing high grade PIN with cribriform pattern in the VP and DLP of 6-month $Bas^{AR\text{-}Pten\text{-}}$ (upper) and $Lum^{AR\text{-}Pten\text{-}}$ tumors (lower). Scale bars correspond to 100 microns.

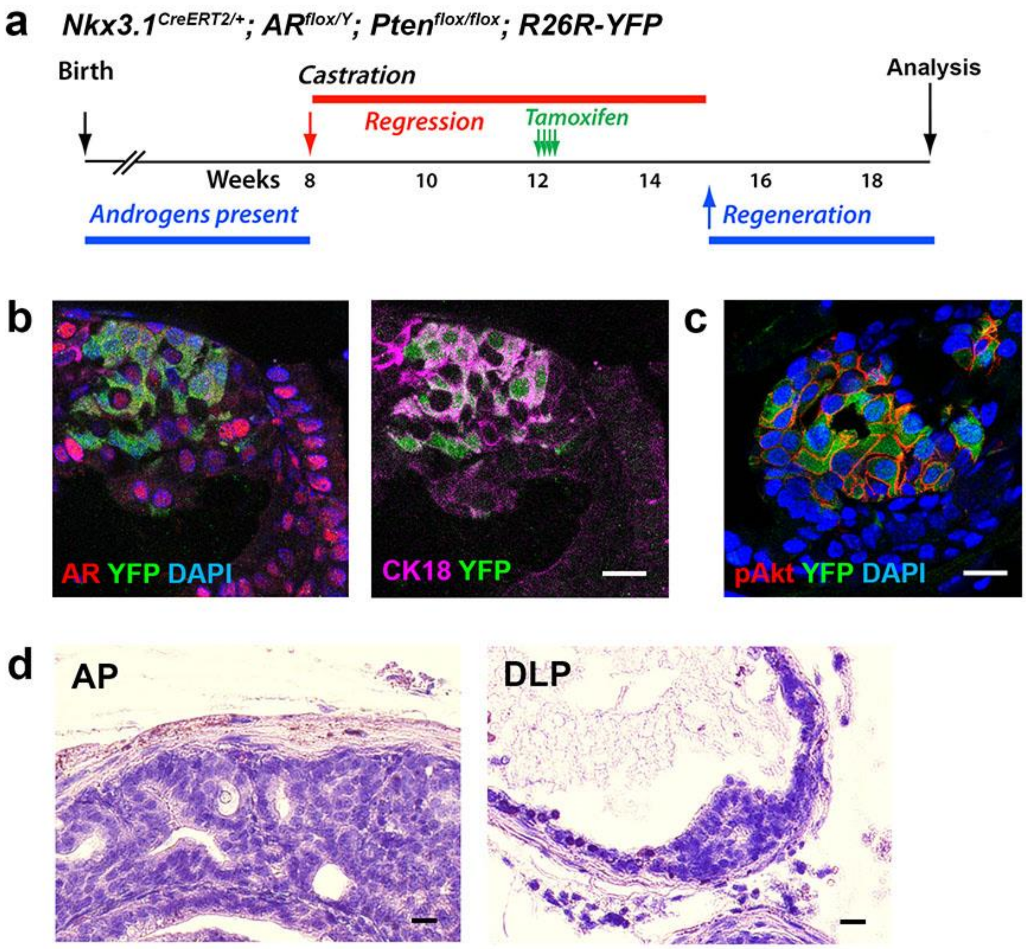

Supplementary Figure 11. Pten loss can override AR loss in CARNs to initiate prostate cancer.

(**a**) Strategy for inducing tumors with AR and Pten double deletion in CARNs. (**b**,**c**) IF staining of AR^- $Pten^-$ CARN-derived tumors showing PIN cells were AR^- and $CK18^+$ (**b**) and express phosphor-Akt (**c**). (**d**) H&E staining showing foci of AR^-Pten^- CARN-derived PIN lesions in the AP and DLP lobes. Scale bars correspond to 20 microns.

a

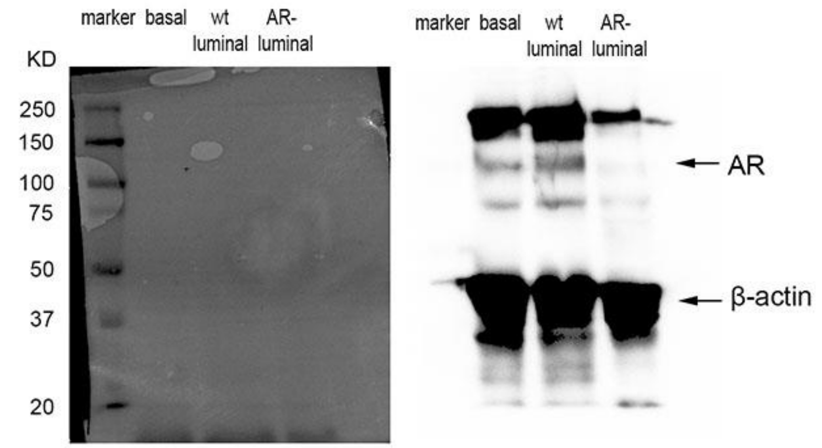

b

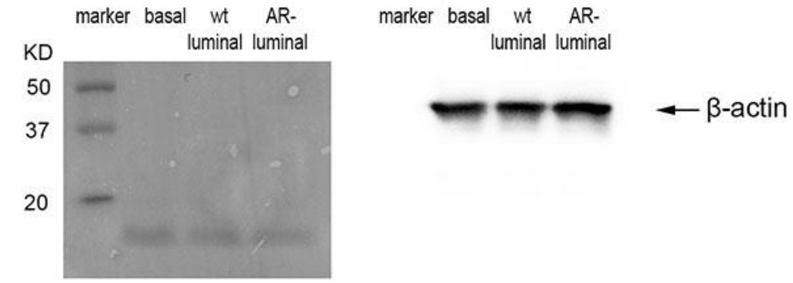

Supplementary Figure 12. Full-size scans of western blots in Fig. 3d.

(**a**) For Fig. 3d upper: western blotting with AR and β–actin antibodies on one membrane. (**b**) For Fig. 3d lower: western blotting with β–actin antibody. Visualization of molecular weight ladder is shown on the left.

Supplementary Table 1. Quantitation of lineage analyses and BrdU incorporation assays in Fig. 1.

**Data for Fig. 1f**			
***Bas**^{YFP}* **2.5m**			
**Mouse ID**	**Total YFP+**	**YFP+AR+**	**Percentage**
#167	1458	786	53.9%
#2345	1105	725	65.6%
#2347	1693	960	56.7%
***Bas**$^{YFP,AR-}$* **2.5m**			
**Mouse ID**	**Total YFP+**	**YFP+AR+**	**Percentage**
#543	2100	465	22.1%
#545	1227	232	18.9%
#548	1588	407	25.6%
***Bas**$^{YFP,AR-}$* **4m**			
**Mouse ID**	**Total YFP+**	**YFP+AR+**	**Percentage**
#150	1030	190	18.4%
#153	1441	295	20.5%
#204	968	262	27.1%
***Bas**$^{YFP,AR-}$* **8m**			
**Mouse ID**	**Total YFP+**	**YFP+AR+**	**Percentage**
#321	1743	354	20.3%
#322	1529	358	23.4%
#325	1580	264	16.7%

**Data for Fig. 1g**						
***Bas**$^{YFP,AR-}$* **2.5m**						
**Mouse ID**	**YFP+AR+**	**YFP+AR+ BrdU+**	**Percentage**	**YFP+AR-**	**YFP+AR- BrdU+**	**Percentage**
#2972	515	8	1.6%	1410	20	1.4%
#2974	228	4	1.8%	890	17	1.9%
#2975	352	5	1.4%	1073	14	1.3%
***Bas**$^{YFP,AR-}$* **8m**						
**Mouse ID**	**YFP+AR+**	**YFP+AR+ BrdU+**	**Percentage**	**YFP+AR-**	**YFP+AR- BrdU+**	**Percentage**
#2120	568	5	0.9%	1860	22	1.2%
#2121	479	7	1.5%	1609	19	1.2%
#2125	470	5	1.1%	1641	16	1.0%

**Data for Fig. 1i**				
Bas ^{YFP} **8m**				
**Mouse ID**	**Total YFP+**	**YFP+CK18+**	**Percentage**	**YFP+CK18+AR+**
#1471	1710	14	0.8%	14
#1480	2073	25	1.2%	25
#1912	1394	20	1.4%	20
Bas $^{YFP,AR-}$ **8m**				
**Mouse ID**	**Total YFP+**	**YFP+CK18+**	**Percentage**	**YFP+CK18+AR+**
#321	2236	7	0.31%	7
#322	1609	4	0.25%	4
#325	1700	6	0.35%	6

Supplementary Table 2. Quantitation of lineage analyses in Fig. 2.

**Data for Fig. 2d & Fig. 2e blue line**						
Bas$^{YFP,AR-}$ **before castration**						
**Mouse ID**	**Total YFP+**	**YFP+AR+**	**YFP+ CK18+**	**Basal AR+ Percentage**	**Lum YFP+ Percentage**	**YFP+ CK18+AR+**
#543	2100	465	0	22.1%	0%	0
#545	1227	232	0	18.9%	0%	0
#548	1588	407	0	25.6%	0%	0
Bas$^{YFP,AR-}$ **regressed**						
**Mouse ID**	**Total YFP+**	**YFP+AR+**	**YFP+ CK18+**	**Basal AR+ Percentage**	**Lum YFP+ Percentage**	**YFP+ CK18+AR+**
#1847	2958	598	0	20.2%	0%	0
#1849	2841	662	0	23.3%	0%	0
#1852	3108	653	0	21.0%	0%	0
Bas$^{YFP,AR-}$ **1 round regeneration**						
**Mouse ID**	**Total YFP+**	**YFP+ CK18-AR+**	**YFP+ CK18+**	**Basal AR+ Percentage**	**Lum YFP+ Percentage**	**YFP+ CK18+AR+**
#319	2692	425	0	15.8%	0%	0
#681	2982	611	1	20.5%	0.03%	1
#685	2850	553	0	19.4%	0%	0
Bas$^{YFP,AR-}$ **3 round regeneration**						
**Mouse ID**	**Total YFP+**	**YFP+ CK18-AR+**	**YFP+ CK18+**	**Basal AR+ Percentage**	**Lum YFP+ Percentage**	**YFP+ CK18+AR+**
#1848	3025	655	10	21.7%	0.33%	10
#2116	2729	630	6	23.1%	0.22%	6
#2117	2831	518	7	18.3%	0.25%	7

**Data for Fig. 2e red line**				
Bas^{YFP} **before castration**				
**Mouse ID**	**Total YFP+**	**YFP+CK18+**	**YFP+CK18+AR+**	**Luminal YFP+ Percentage**
#167	1458	0	0	0%
#2345	1105	0	0	0%
#2347	1693	0	0	0%
Bas^{YFP} **regressed**				
**Mouse ID**	**Total YFP+**	**YFP+CK18+**	**YFP+CK18+AR+**	**Luminal YFP+ Percentage**
#365	2278	0	0	0%
#370	2764	0	0	0%
Bas^{YFP} **1 round regeneration**				

**Mouse ID**	**Total YFP+**	**YFP+CK18+**	**YFP+CK18+AR+**	**Luminal YFP+ Percentage**
#366	2489	2	2	0.08%
#367	2214	1	1	0.05%
#767	3540	4	4	0.11%
Bas^{YFP} **3 round regeneration**				
**Mouse ID**	**Total YFP+**	**YFP+CK18+**	**YFP+CK18+AR+**	**Luminal YFP+ Percentage**
#1892	3055	23	23	0.75%
#1894	2263	20	20	0.88%
#2349	2672	28	28	1.05%

Supplementary Table 3. Quantitation of lineage analyses and BrdU incorporation assays in Fig. 3.

**Data for Fig. 3f**						
Lum$^{YFP,AR-}$ **9w (BrdU for 1w)**						
**Mouse ID**	**AR+ luminal**	**BrdU+AR+ luminal**	**Percentage**	**AR- luminal**	**BrdU+AR- luminal**	**Percentage**
#2789	2272	86	3.8%	369	50	13.6%
#2793	1943	85	4.4%	246	28	11.4%
#2843	2435	110	4.5%	380	56	14.7%
#3068	2329	75	3.2%	411	38	9.2%
Lum$^{YFP,AR-}$ **10w (BrdU for 2w)**						
**Mouse ID**	**AR+ luminal**	**BrdU+AR+ luminal**	**Percentage**	**AR- luminal**	**BrdU+AR- luminal**	**Percentage**
#2787	1021	61	6.0%	288	48	16.7%
#2788	1996	124	6.2%	450	64	14.2%
#6456	1887	134	7.1%	404	76	18.8%
Lum$^{YFP,AR-}$ **12w (BrdU for 2w)**						
**Mouse ID**	**AR+ luminal**	**BrdU+AR+ luminal**	**Percentage**	**AR- luminal**	**BrdU+AR- luminal**	**Percentage**
#2307	1745	87	5.0%	308	10	3.2%
#2308	1520	94	6.2%	335	15	4.5%
#3072	1028	48	4.7%	290	18	6.2%
#6457	1982	125	6.3%	618	32	5.2%
#6458	1994	93	4.7%	529	27	5.1%
Lum$^{YFP,AR-}$ **5m (BrdU for 2w)**						
**Mouse ID**	**AR+ luminal**	**BrdU+AR+ luminal**	**Percentage**	**AR- luminal**	**BrdU+AR- luminal**	**Percentage**
#2310	1600	90	5.6%	378	27	7.1%
#2312	1307	64	4.9%	240	14	5.8%
#3069	1588	105	6.6%	307	19	6.2%
#3070	1471	100	6.8%	265	17	6.4%
Lum$^{YFP,AR-}$ **8m (BrdU for 2w)**						
**Mouse ID**	**AR+ luminal**	**BrdU+AR+ luminal**	**Percentage**	**AR- luminal**	**BrdU+AR- luminal**	**Percentage**
#1255	930	48	5.2%	178	9	5.1%
#1256	1146	63	5.5%	249	16	6.4%
#1259	1951	98	5.0%	389	20	5.1%

Data for Fig. 3i					
***Lum**$^{YFP,AR-}$* **10w**					
**Mouse ID**	**Total luminal**	**YFP+ luminal**	**AR- luminal**	**YFP+ Lum Percentage**	**AR- Lum Percentage**
#2505	627	85	97	13.6%	15.5%
#2507	844	104	137	12.3%	16.2%
#2841	551	100	119	18.1%	21.6%
#4397	1156	229	272	19.8%	23.5%
***Lum**$^{YFP,AR-}$* **12w**					
**Mouse ID**	**Total luminal**	**YFP+ luminal**	**AR- luminal**	**YFP+ Lum Percentage**	**AR- Lum Percentage**
#1048	1709	239	264	14.0%	15.4%
#1181	1255	216	252	17.2%	20.1%
#1185	2143	268	281	12.5%	13.1%
***Lum**$^{YFP,AR-}$* **5m**					
**Mouse ID**	**Total luminal**	**YFP+ luminal**	**AR- luminal**	**YFP+ Lum Percentage**	**AR- Lum Percentage**
#1444	1884	373	418	19.8%	22.2%
#1827	930	129	136	13.9%	14.6%
#1828	1400	228	262	16.3%	18.7%
***Lum**$^{YFP,AR-}$* **8m**					
**Mouse ID**	**Total luminal**	**YFP+ luminal**	**AR- luminal**	**YFP+ Lum Percentage**	**AR- Lum Percentage**
#1445	2020	261	319	12.9%	15.8%
#2790	1696	263	293	15.5%	17.3%
#2842	1305	193	201	14.8%	15.4%
#3525	1082	110	119	10.2%	11.0%

Supplementary Table 4. Quantitation of lineage analyses and BrdU incorporation assays in Fig. 5.

**Data for Fig. 5e**						
Lum$^{YFP,AR-}$ **BrdU 12d during regeneration**						
**Mouse ID**	**AR+ luminal**	**BrdU+AR+ luminal**	**Percentage**	**AR- luminal**	**BrdU+AR- luminal**	**Percentage**
#3071	1408	584	41.5%	378	133	35.2%
#3074	859	407	47.4%	175	77	44.0%
#3075	948	349	36.8%	295	119	40.3%

**Data for Fig. 5f**					
Lum$^{YFP,AR-}$ **10w**					
**Mouse ID**	**Total luminal**	**YFP+ luminal**	**AR- luminal**	**YFP+ Lum Percentage**	**AR- Lum Percentage**
#2505	627	85	97	13.6%	15.5%
#2507	844	104	137	12.3%	16.2%
#2841	551	100	119	18.1%	21.6%
Lum$^{YFP,AR-}$ **castrated**					
**Mouse ID**	**Total luminal**	**YFP+ luminal**	**AR- luminal**	**YFP+ Lum Percentage**	**AR- Lum Percentage**
#2371	1396	250	279	17.9%	20.0%
#1180	912	152	183	16.7%	20.1%
#1184	880	139	160	15.8%	18.2%
Lum$^{YFP,AR-}$ **regenerated**					
**Mouse ID**	**Total luminal**	**YFP+ luminal**	**AR- luminal**	**YFP+ Lum Percentage**	**AR- Lum Percentage**
#1829	960	124	141	12.9%	14.7%
#2794	1423	292	336	20.5%	23.6%
#2795	908	171	203	18.8%	22.4%

Supplementary Table 5. Quantitation of lineage analyses and BrdU incorporation assays in Fig. 6.

Data for Fig. 6d			
Lum^{YFP} **castrate, induce, regenerated**			
**Mouse ID**	**Single YFP+ cells**	**Clustered YFP+ cells**	**Clustered Percentage**
#2024	70	247	77.9%
#2030	64	168	72.4%
#2042	83	175	67.8%
$Lum^{YFP,AR-}$ **castrate, induce, regenerated**			
**Mouse ID**	**Single YFP+ cells**	**Clustered YFP+ cells**	**Clustered Percentage**
#1669	68	8	10.5%
#2027	58	9	13.4%
#2029	111	12	9.8%

Data for Fig. 6h			
Lum^{YFP} **castrate, induce, regenerate with BrdU for 12d**			
**Mouse ID**	**YFP+**	**YFP+BrdU+**	**Percentage**
#2441	87	47	54.0%
#2443	95	60	63.2%
#2444	113	58	51.3%
$Lum^{YFP,AR-}$ **castrate, induce, regenerate with BrdU for 12d**			
**Mouse ID**	**YFP+AR-**	**YFP+AR-BrdU+**	**Percentage**
#2510	72	35	48.6%
#2512	88	49	55.7%
#3073	73	40	54.8%

Supplementary Table 6. Primary antibodies used in this study.

Antibodies for immunofluorescence and immunohistochemistry			
**Antigen**	**Supplier**	**Ig type**	**Dilution**
AR	Sigma #A9853	rabbit IgG	1:400
αPKC	Santa Cruz #sc-216	rabbit IgG	1:200
Cleaved Caspase 3	Cell Signaling #9661	rabbit IgG	1:500
CK5	Covance #PRB-160P	rabbit IgG	1:500
CK18	Abcam #ab668	mouse IgG1	1:100
E-Cadherin	BD Biosciences #610181	mouse IgG2a	1:500
Ki67	DakoCytomation #M7249	rat IgG2a	1:600
Nkx3.1	Kim et al. (2002) PNAS 99: 2884-2889	rabbit IgG	1:2000
p63	Santa Cruz #sc-8343	rabbit IgG	1:50
p63	GeneTex #GTX102425	rabbit IgG	1:1000
Phospho-Akt	Cell Signaling #3787	rabbit IgG	1:50
YFP	Abcam #13970	chick IgY	1:2000

Antibodies for flow cytometry		
**Antibody**	**Supplier**	**Dilution**
Sca-1-PE-Cy7	Biolegend clone E13-161.7 #122513	1:500
CD49f-PE	eBiosciences clone eBioGoH3 #12-0495	1:300
Ter119-eFluor450	eBiosciences clone Ter-119 #48-5921	1:250
CD31-eFluor450	eBiosciences clone 390 #48-0311	1:250
CD45-eFluor450	eBiosciences clone 30-F11 #48-0451	1:250

Supplementary Table 7. List of primer sequences for quantitative real-time PCR.

Gene	Primer Sequences
Actb	TGTGAGATCCACGGAAACAG
	ACATAGCCGGAACCTACGTG
Ccnd1	AAGGCTTTTCCCAGTCCTTC
	CCCTCATCTAGCGTCTCAGG
Myc	AGTGCTGCATGAGGAGACAC
	GGTTTGCCTCTTCTCCACAG
Cdkn1a	GTCTGAGCGGCCTGAAGAT
	TCTGCGCTTGGAGTGATAGA
Krt5	GAGATCGCCACCTACAGGAA
	TCCTCCGTAGCCAGAAGAGA
Krt8	GCACTCAGGAGAAGGAGCAG
	CTCCACTTGGTCTCCAGCAT
Krt14	ACCATGCAGAACCTGGAGAT
	CAGTAACGGCCTTTGGTCTC
Krt18	AAATCGAGGCACTCAAGGAA
	AATCTGGGCTTCCAGACCTT
Nkx3-1	GGAGGACCCACCAAGTATCC
	CACTTGCTAAGTCCCCTGGA
Trp63	GTAGAAGGGAACAGCCATGC
	TTGTGAATTCAGTGCCAACC
